# Phenomic profiles and disease patterns of 1.9 million participants from Our Future Health

**DOI:** 10.1038/s41591-026-04602-4

**Published:** 2026-09-10

**Authors:** Vincent J. Straub, Stefania Benonisdottir, Giovanni Scotti Bentivoglio, Neil Wary, Robert Campbell, Augustine Kong, Melinda C. Mills

**Affiliations:** 1 Leverhulme Centre for Demographic Science, Nuffield Department of Population Health, University of Oxford and Nuffield College, Oxford, UK; 2 Institute of Physical Sciences, University of Iceland, Reykjavík, Iceland; 3 Department of Economics, Econometrics and Finance, University of Groningen, Groningen, the Netherlands; 4 Department of Genetics, University Medical Centre Groningen, Groningen, the Netherlands

**Keywords:** Medical research, Databases, Epidemiology

## Abstract

Our Future Health is a prospective study aiming to recruit 5 million UK-resident adults to enable discovery and translation of disease prevention, detection and treatment approaches. So far, more than 2.5 million have enrolled, and baseline phenotypic data are available for >1.9 million participants. Here we provide an assessment of phenotypes—self-reported health-related behaviors, geolocation, diagnoses and medication, in- and outpatient visits, cancer registry and cause of death—and comparison of disease patterns against national estimates and the UK Biobank cohort. Sociodemographic, lifestyle and health-related characteristics reflected UK population patterns; all but one minority ethnic group and the most socioeconomically deprived groups were underrepresented. The prevalence of several major self-reported conditions, particularly mental health conditions such as depression and anxiety, was higher than national estimates and directionally concordant with the UK Biobank (*r* = 0.78). Associations with known clinical correlates replicated across both cohorts (*r* = 0.80). Medication-use patterns and cancer prevalence followed expected age-related gradients, with lower lung cancer rates than national data. As recruitment progresses, electronic health records can help specify disease patterns and systematically assess biases.

## Abstract (web summary)

An in-depth phenomic analysis of health and disease patterns from 1.9 million participants of the Our Future Health cohort reports the prevalence of health conditions and medication-use patterns compared with national UK estimates and the UK Biobank.

## Main

Healthcare systems face mounting challenges to building a healthier future for their populations, including a growing burden of chronic disease driven by aging, lifestyle changes and environmental exposures, alongside persistent health inequities and reliance on fragmented data^1^. Biomedical research increasingly involves combining diverse data to consider the interplay of multiomics data, lifestyle factors, social determinants and the environment to assess population health and identify populations at higher risk of disease^2^. Large-scale cohort studies and biobanks^3–7^, which systematically collect, store and manage health and biological data over time, have come to play a leading role in shaping developments in biomedical research^8^. Through deep data integration, these resources provide scientific insights into the determinants of health and disease^9^ as well as offering opportunities to address clinical and public health challenges^10^.

In the UK, an established health and biomedical research infrastructure^11^ has facilitated the successes of existing cohorts^3,12,13^. These resources have catalyzed a broad array of clinical and translational research, in addition to connecting researchers and participants; 1 in 30 people in the UK are estimated to take part in cohort studies^14^. Yet, existing volunteer-based studies are not always representative of their target population^15^; findings may not always extrapolate to all communities owing to a lack of diversity^16,17^, and, despite their depth, existing cohorts may not be sufficient in size to power discovery research, especially on less common diseases. Alongside these known data limitations, there are also several well-studied sampling and surveying issues that can impact the composition of cohorts, most notably, various selection biases (see box 1 in the Supplementary Information for more details). Such biases can confound interpretation of analysis results and may reduce the generalizability of exposure–disease associations^18^, as has long been discussed^19,20^.

**Fig. 1:**
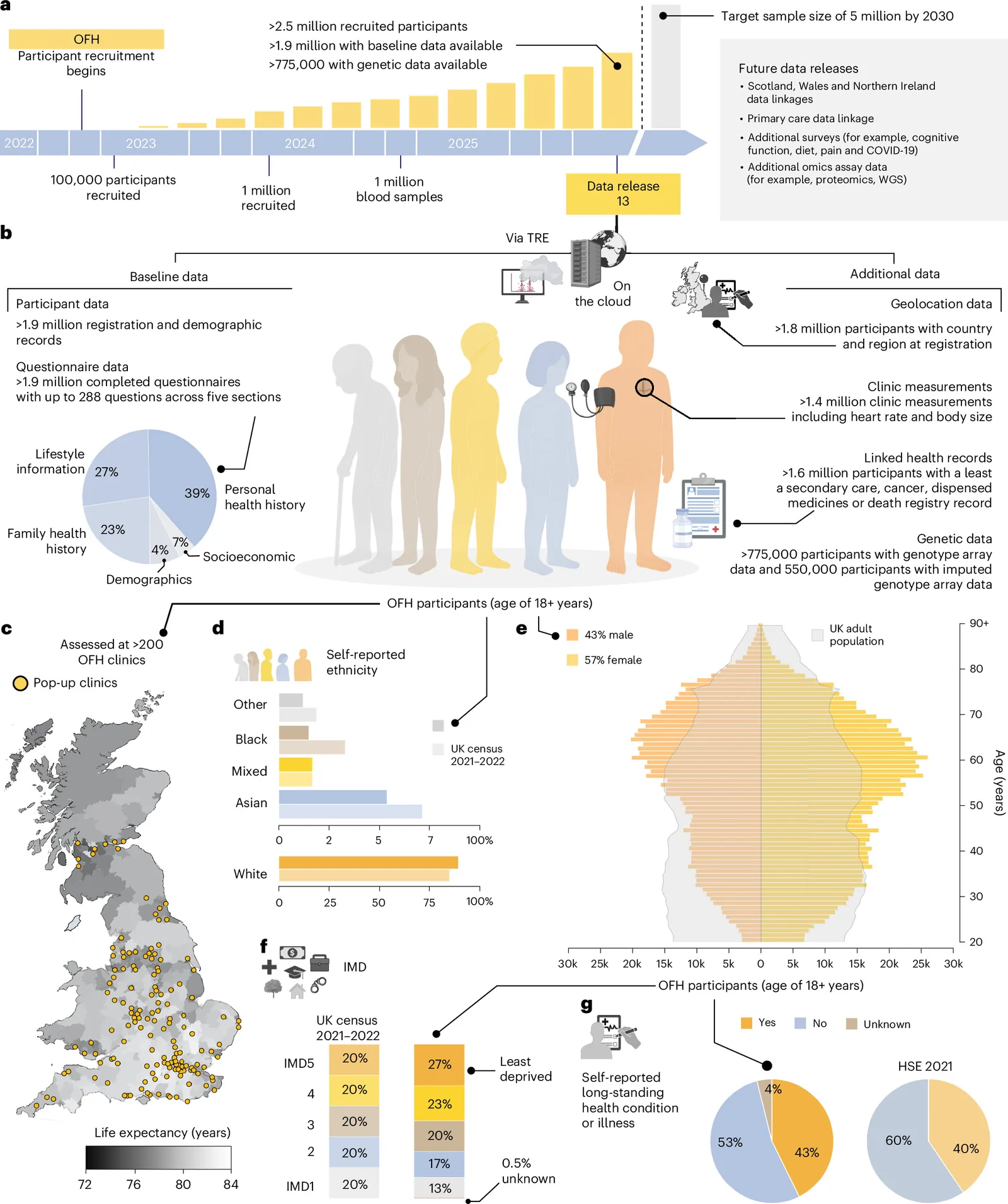
Overview of OFH data and cohort. **a**, The timeline of data collection and data available in data release 13, released on 11 December 2025. **b**, An overview of available data in release 13. **c**, Locations of OFH pop-up clinics across the UK, overlaid on a regional heat map of life expectancy. **d**, The self-reported ethnicity composition compared with the 2021–2022 UK Census. **e**, The population age–sex structure compared with the mid-2023 population estimates for Great Britain; percentages for Great Britain are multiplied by total number of OFH participants to report the two quantities on the same scale. **f**, The distribution of IMD quintiles compared with the UK Census, restricted to participants with IMD data available via linked health records (*N* = 1,370,712). **g**, The distribution of self-reported long-standing health condition or illness compared with HSE 2021 (ref. ^54^). Unless otherwise stated, *N* = 1,900,193 participants. Extended footnotes are provided in Supplementary Information. Figure created in BioRender; Straub, V. https://biorender.com/5p221tm (2026). Map in **c** created using data from the ONS under an Open Government Licence.

**Extended Data Fig. 1:**
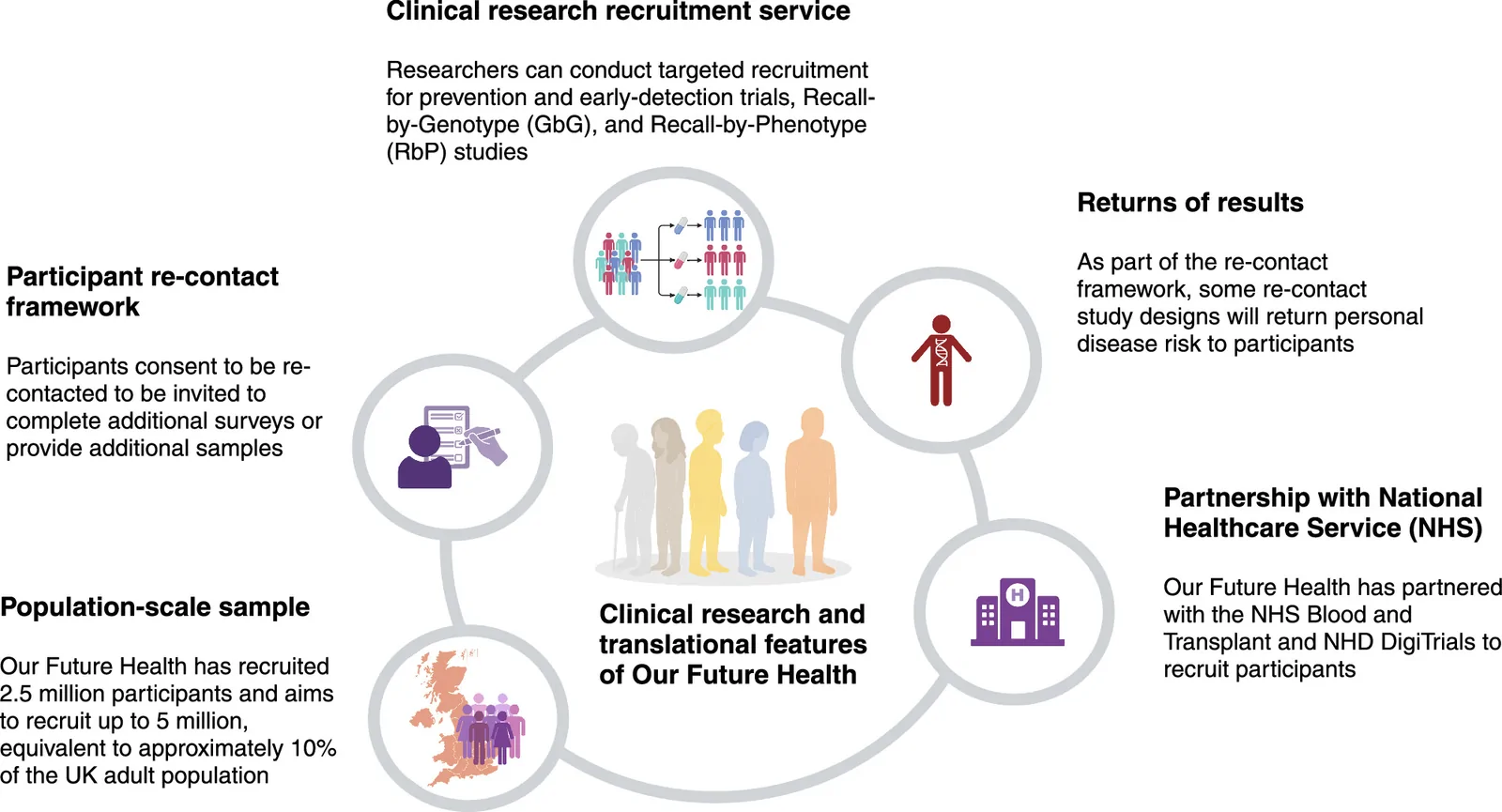
Translational research features of Our Future Health. Summary of translation features of Our Future Health including its sample size; participant re-contact framework; clinical research recruitment service (https://research.ourfuturehealth.org.uk/clinical-research-recruitment/); return of results to participants; and its recruitment partnership with the NHS. NHS, National Healthcare Service. Figure created in BioRender; Straub, V. https://biorender.com/3of9x7l (2026).

Our Future Health (OFH) is a recently established prospective cohort study supported by the UK Government, industry and charity sectors (see box 1 in ref. ^21^) to enable the discovery and translation of more effective approaches to disease prevention, detection and treatment (Fig. 1). Its ambition is to recruit a sample of 5 million UK-resident adults from a target population of *n* = 53,108,969 (approximately the UK adult population in 2021–22), which is reflective of the UK population in terms of age, sex and ethnicity. If OFH achieves its sample ambition (Extended Data Table 1), its cohort will cover approximately 10% of the UK adult population. Since recruitment began in late 2022, more than 2.5 million individuals have registered to participate in OFH (Extended Data Table 2), and baseline phenotypic data are currently available on >1.9 million participants (Fig. 1a). This makes OFH, at the time of writing, the largest multi-ethnic cohort and largest cohort of young people (18–40 years of age), working-age populations and people over 60 years of age in the world^22^. Although data collection is ongoing, the scale and population coverage means OFH already offers opportunities to advance health research and genomic medicine^21^. In terms of clinical potential, additional features of OFH, specifically its clinical research recruitment service^23^ (Extended Data Fig. 1), make it well-positioned to help address traditional translational bottlenecks often faced by biobanks^9^, helping to differentiate it from other existing national, large-scale initiatives^15,24–26^.

Here, we provide an assessment of OFH phenotypic data at its halfway recruitment goal, focusing on phenotypes relevant to clinical medicine and public health^23^. Our primary aim was to explore the phenomic profiles of participants, compared against national estimates and established large-scale biobanks, most relevantly the UK Biobank^3^ (UKB; ‘Study populations’ in Methods). An additional, secondary aim was to investigate the representativeness of participant responses and identify potential sources of bias (see Box 1 in the Supplementary Information for more details) that may require further investigation to improve the robustness of future research using OFH.

## Results

### The OFH cohort

**Extended Data Fig. 2:**
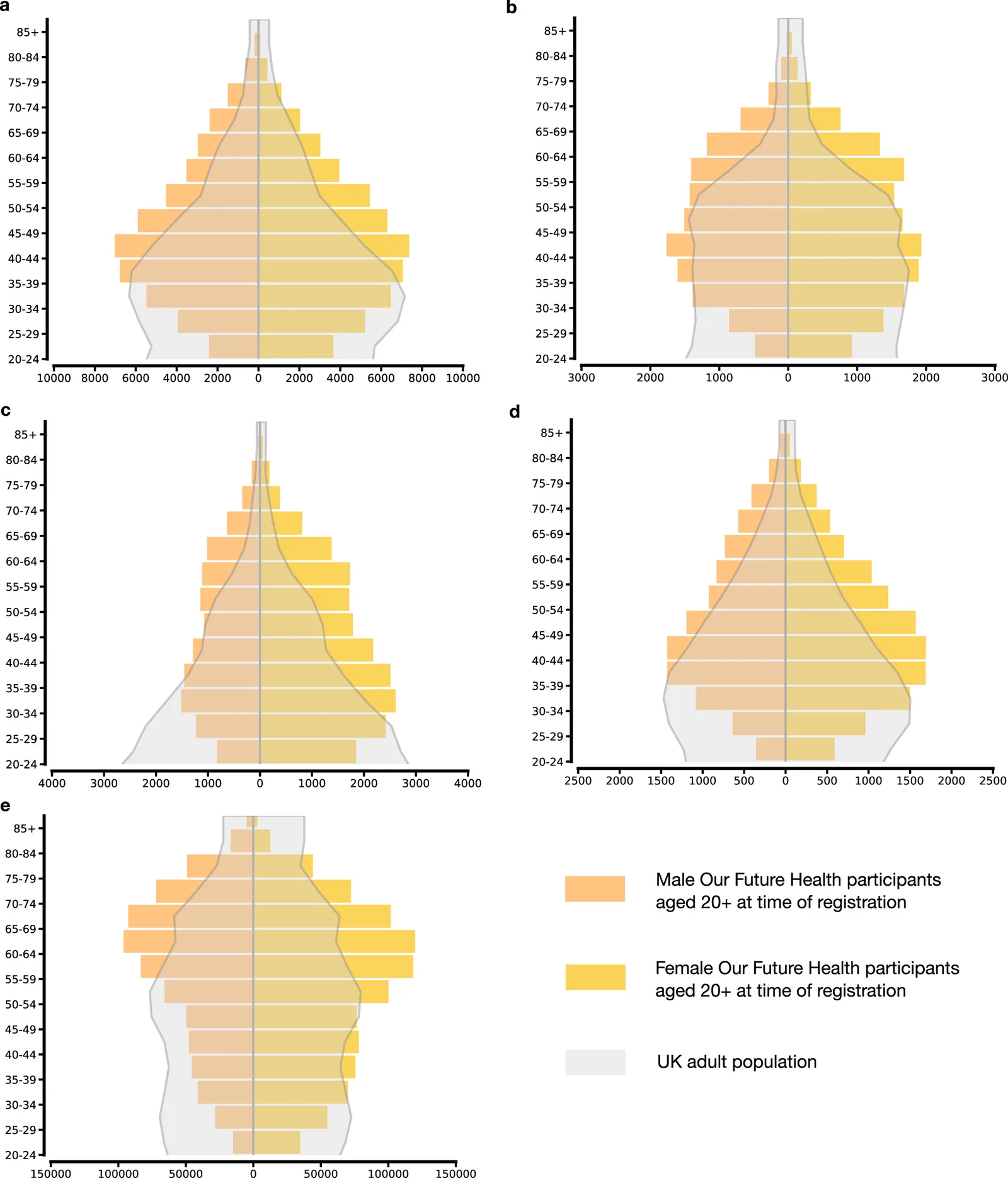
Population age–sex structure compared with the 2021/2022 UK Census by major self-reported ethnic group. Percentages for UK are multiplied by total number of OFH participants by ethnic group to report the two quantities on the same scale. **a**, Asian (Asian Bangladeshi, Asian Indian, Asian Pakistani, Chinese, Other Asian). **b**, Black (Black African, Black Caribbean, Other Black). **c**, Mixed. **d**, Other (Arab, Any other). **e**, White (White British, Irish, Polish, Other White). UK, United Kingdom; OFH, Our Future Health.

The sociodemographic composition of the cohort broadly reflected that of the UK adult population, while also revealing systematic differences informative for interpretation and ongoing recruitment (Fig. 1c–g). The age–sex structure broadly mirrored that of the UK population but included a higher proportion of female participants (57% versus 43%), similar to the UKB^15^ and the Estonian Biobank^7^, and fewer younger adults, with patterns varying by self-reported ethnic group (Extended Data Fig. 2). Although this means that non-white participants and younger age-groups are still underrepresented compared with the UK population, given the size of OFH, the absolute impact of their representation is substantial. For instance, there are alone more than 40,000 young participants (aged 18–39 years) who identify as Asian British or any other Asian or Asian British background (Supplementary Table 10.2), more than in any existing UK-based cohort^18^. In terms of deprivation, participants were underrepresented in the most deprived index of multiple deprivation (IMD) quintile (IMD 1: 13% versus 20% nationally) and overrepresented in the least deprived quintile (IMD 5: 27% versus 20%), whereas intermediate quintiles were more closely aligned with population estimates (Fig. 1f). As for self-reported health, a slightly higher proportion of participants reported having a long-standing health condition or illness compared with estimates from the 2021 Health Survey England (HSE; ‘Study populations’ in Methods) (43% versus 40%), although the majority reported no such condition (53% versus 60%) (Fig. 1g). Given the OFH recruitment strategy^23^, which involved a phased roll-out beginning in comparatively more deprived areas of England, it is important to note that the characteristics of recruited participants vary over time and across data releases (Extended Data Table 2).

### Geographic distribution

**Fig. 2:**
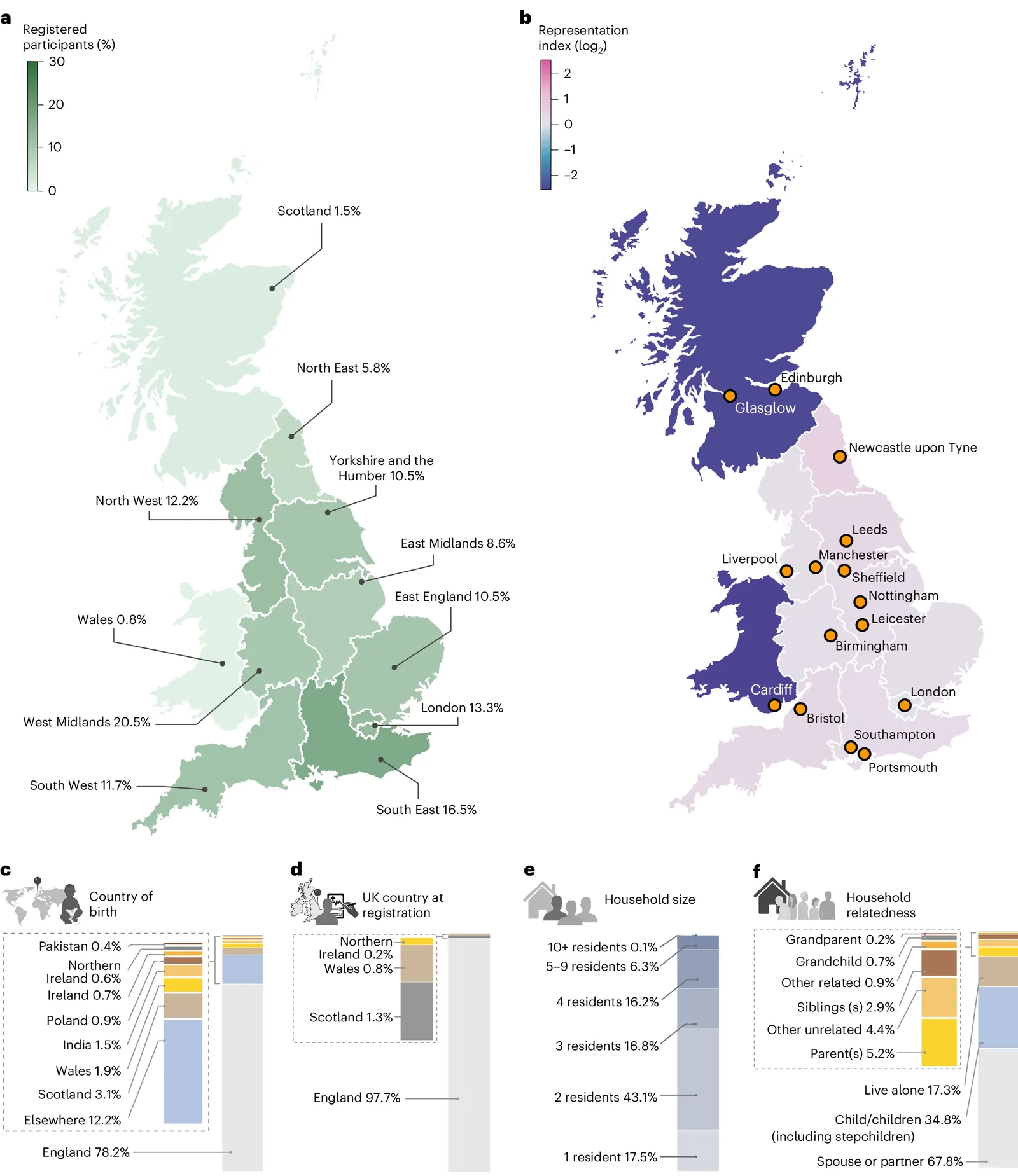
Geographic distribution and self-reported household characteristics by OFH participants. **a**, The frequency of participants registered per self-reported region of registration for participants with available geolocation data (*N* = 1,841,458). **b**, The regional representation of participants with available geolocation data relative to the UK population. A log_2_ representation index compares the proportion of enrolled participants residing in each UK region with the corresponding proportion of the UK population from the 2021–2022 Census; values above zero indicate overrepresentation, and values below zero indicate underrepresentation. Regions are shaded according to the magnitude and direction of deviation, and major cities are shown for reference. **c**, The self-reported country of birth. **d**, The self-reported country at registration. **e**, The self-reported household size. **f**, The self-reported household relatedness (multiple categories could be selected; therefore, percentages do not sum to 100%). *N* = 1,900,495 unless otherwise stated. Extended footnotes are provided in the Supplementary Information. Icons in **c**–**f** created in BioRender; Straub, V. https://biorender.com/3log4y6 (2026). Maps created using data from the ONS under an Open Government Licence.

The influence of the OFH recruitment strategy, alongside existing patterns of UK population demography, can be seen in the spatial distribution of participants, which varied across UK regions (Fig. 2a,b). Importantly, enrollment patterns are consistent with the phased roll-out of recruitment (Methods). In addition, the recruitment strategy prioritized invitation to participants within approximately a 5-mile radius of newly opened appointment centers^23^, which are concentrated in urban areas (Fig. 1c), contributing to geographically clustered enrollment. At registration, 97.7% of participants reported residing in England (Fig. 2d), further reflecting the timing and geographic focus of recruitment. The pattern of many participants reporting living with multiple individuals per household (Fig. 2e) may, in part, also reflect the recruitment strategy, which involved inviting both individuals and households^23^; thereby potentially enabling the enrollment of multiple related participants from the same household. Consistent with this, the most reported household relationships were living with a husband, wife or partner (67.8%) (Fig. 2f).

### Comparison of lifestyle, health and anthropometric measures

Anthropometric, lifestyle and health-related characteristics of OFH participants were broadly similar to national estimates from the 2021 HSE (‘Study populations’ in Methods), although with several notable differences (Table 1). Mean body mass index (BMI) was comparable between cohorts for both male (27.7 kg m^−2^ in OFH versus 27.6 kg m^−2^ in HSE) and female participants (27.0 kg m^−2^ versus 27.4 kg m^−2^), and the overall distribution of BMI categories was similar, although normal overweight was marginally more prevalent among both male and female OFH participants. Waist circumference was lower in OFH participants than in HSE for both sexes, whereas height and weight distributions were broadly comparable (Table 1). Notably, waist circumference was measured only for surveyed HSE respondents who attended the nurse appointment (*N* = 1,663), and overweight, obese BMI categories are slightly overrepresented in this subsample compared with the whole HSE sample. Relatedly, there is a higher incidence of missing data for HSE BMI status, which may explain several of these differences (see section 2.4 in the Supplementary Information for details).

**Table 1:** Comparison of anthropometric, lifestyle and health-related measures, by self-reported sex registered at birth, for OFH participants compared with HSE (2021)

Measure	Male				Female			
	OFH (*N* = 810,493)		HSE (*N* = 2,647)		OFH (*N* = 1,088,419)		HSE (*N* = 3,127)	
	Mean	s.d.	Mean	s.d.	Mean	s.d.	Mean	s.d.
Anthropometrics^a^								
BMI (kg m^−2^)	27.68	4.98	27.64	4.86	27.04	5.99	27.44	5.99
Height (cm)	176.40	7.36	175.90	7.03	163.17	6.78	162.29	6.59
Weight (kg)	86.12	15.73	85.44	15.98	71.97	16.22	72.27	16.30
Waist circumference	96.32	13.28	99.22	13.25	85.37	14.33	89.98	14.15
Smoking initiation								
Age started smoking regularly	17.44	4.15	17.27	4.04	17.54	4.22	17.73	5.47
	*N*	%	***N***	%	*N*	%	*N*	%
Alcohol consumption								
Daily or almost daily	101,249	12.49	337	12.73	75,888	6.97	269	8.60
3–4 times a week	185,164	22.85	452	17.08	177,281	16.29	380	12.15
1–2 a week	225,399	27.81	722	27.28	271,946	24.99	718	22.96
1–3 times a month	111,593	13.77	296	11.18	188,594	17.33	455	14.55
Special occasions only	106,507	13.14	357	13.49	243,057	22.33	604	19.32
Never	76,664	9.46	457	17.26	128,054	11.77	686	21.94
Prefer not to answer/missing	3,917	0.48	24	0.91	3,599	0.33	15	0.48
Smoking status								
Current	59,838	7.38	350	13.22	71,437	6.56	330	10.55
Previous	253,054	31.22	635	23.99	300,090	27.57	689	22.03
Never	482,107	59.48	1,638	61.88	699,678	64.28	2,095	67.00
Prefer not to answer/missing	15,494	1.91	22	0.83	17,214	1.58	13	0.42
E-cigarette status (vaping)								
Ever used	130,807	16.14	483	18.25	175,697	16.14	421	13.46
Never used	672,901	83.02	2,142	80.92	905,336	83.18	2,694	86.15
Prefer not to answer/missing	6,785	0.83	22	0.83	7,386	0.68	12	0.38
Self-reported health status								
Excellent^b^	118,799	14.66	951	35.93	156,187	14.35	1,095	35.02
Good	444,868	54.89	1,106	41.78	603,034	55.4	1,272	40.68
Fair	200,416	24.73	439	16.58	259,210	23.82	542	17.33
Poor	42,137	5.20	144	5.44	64,255	5.90	215	6.88
Prefer not to answer/missing	4,273	0.52	7	0.26	5,733	0.52	3	0.10
Physical activity								
≥16 times a month walk for at least 10 min	629,420	77.66	1,194	45.11	848,338	77.94	1,584	50.66
<16 times a month walk for at least 10 min	158,077	19.5	992	37.48	210,324	19.32	1,082	34.60
Prefer not to answer/missing	22,996	2.84	463	17.49	73,099	6.72	464	14.82
BMI status								
Underweight	2,632	0.42	25	0.94	11,962	1.5	54	1.73
Normal	183,951	29.12	724	27.35	333,065	41.71	1,068	34.15
Overweight	287,766	45.55	1,099	41.52	256,713	32.15	949	30.35
Obese	156,929	24.84	646	24.40	196,072	24.56	776	24.82
Missing	453	0.07	153	5.78	621	0.08	280	8.95

^a^Limited to OFH participants who completed their in-person clinic measurements appointment (*N* = 631,728 self-reported male participants; *N* = 798,431 self-reported female participants) and HSE participants who attended the nurse appointment (*N* = 1,663). ^b^Recorded as ‘Very good’ in HSE; see Extended Data Table 2 for a comparison of the difference data collection methods in HSE and OFH. Additional comparisons by age-group and self-reported ethnicity are available in Supplementary Tables 4.1 and 4.2. See ref. ^54^ for further information about HSE data. Extended footnotes are provided in the Supplementary Information.

Health-related behaviors differed systematically between cohorts. Current smoking was less commonly reported in OFH than in HSE among both male (7.4% in OFH versus 13.2% in HSE) and female participants (6.6% versus 10.6%), as was never smoking, albeit with smaller absolute differences across cohorts (59.5% versus 61.9% for male participants and 64.3% versus 67% for female participants). E-cigarette use was similar across cohorts, with >80% of participants reporting never having used vaping products. Patterns of alcohol consumption differed modestly, with a noticeably higher proportion of OFH male and female participants reporting frequent consumption (three to four times a week), particularly among male participants (22.9% versus 17% in HSE). Physical activity levels were higher in OFH, with approximately three quarters of both male and female participants reporting walking for at least 10 min on 16 or more occasions per month, compared with around half of HSE respondents. Self-rated health status also differed between cohorts. Although the majority of OFH participants reported their health as ‘good’ or ‘fair’ (79% combined), fewer reported ‘excellent’ health (14%) compared with HSE respondents (36%), and a slightly higher proportion reported ‘fair’ or ‘poor’ health (Table 1). We note, however, that slight differences in questionnaire wording and response options between OFH and HSE may contribute to these discrepancies (Supplementary Information Table 1). Stratified analyses indicated that differences in anthropometric and lifestyle measures between OFH and HSE further varied by age group and self-reported ethnicity (Supplementary Tables 4.1 and 4.2). Additional socioeconomic characteristics of OFH participants further contextualize these findings (Table 2). In terms of available clinic measurements, across individuals, mean systolic and diastolic blood pressure on the second measurement were 126.6 and 80.1 mm Hg, respectively, with higher values observed in male than female participants, whereas female participants exhibited modestly higher mean heart rate values (Supplementary Table 4.3).

**Table 2:** Socioeconomic characteristics of OFH participants (data release 12)

	Total (*N* = 1,900,495)		Male^a^ (*N* = 810,707)		Female^b^ (*N* = 1,088,628)	
	*N*	%	*N*	%	*N*	%
Annual household income						
<£18,000	180,853	9.5	63,059	7.8	117,475	10.8
£18,000 to £30,999	288,336	15.2	117,296	14.5	170,913	15.7
£31,000 to £51,999	389,355	20.5	171,404	21.1	217,821	20
£52,000 to £100,000	493,125	25.9	225,254	27.8	267,757	24.6
>£100,000	239,289	12.6	118,815	14.7	120,421	11.1
Do not know/prefer not to answer	309,737	16.3	114,879	14.2	194,241	17.8
Qualifications (highest level)^b^						
College or university degree	981,868	51.7	410,662	50.7	570,777	52.4
A levels/AS levels/BTEC or equivalent	312,572	16.4	134,479	16.6	177,953	16.3
O levels, GCSEs or equivalent	294,391	15.5	118,996	14.7	175,307	16.1
CSEs or equivalent	80,070	4.2	36,474	4.5	43,577	4
NVQ, HND or HNC or equivalent	67,805	3.6	36,148	4.5	31,626	2.9
Other professional qualifications, for example, nursing or teaching	43,251	2.3	16,949	2.1	26,285	2.4
None of the above	91,814	4.8	43,914	5.4	47,633	4.4
Prefer not to answer/missing	28,924	1.5	13,085	1.6	15,470	1.4
Employment status (first answer) ^c^						
Full or part-time student	59,246	3.1	17,674	2.2	41,514	3.8
In paid employment or self-employed	1,165,432	61.3	490,166	60.5	674,817	62
Looking after home and/or family	66,428	3.5	8,776	1.1	57,623	5.3
On paid leave	10,504	0.6	1,104	0.1	9,394	0.9
Retired	571,458	30.1	276,582	34.1	294,671	27.1
Unable to work because of sickness or disability	54,787	2.9	18,961	2.3	35,724	3.3
Unemployed	35,115	1.8	15,058	1.9	20,011	1.8
Unpaid or voluntary work/unpaid carer	73,376	3.9	23,032	2.8	50,306	4.6
None of the above/prefer not to answer	16,992	0.9	7,056	0.9	9,605	0.9

Selected characteristics of participants in data release 12 (released on 17 September 2025) are presented, used in most of the analyses in this study; see OFH participant data (https://ourfuturehealth.gitbook.io/our-future-health/data/data-releases/2025-data-releases/release-12) for characteristics of participants in data release 13 (released on 11 December 2025), which include data for an additional *n* = 29,257 participants. ^a^Based on reported sex assigned at birth. Participants who reported ‘other’, ‘intersex’ or ‘prefer not to answer’ are not shown. ^b^UK-specific qualifications (or equivalent): A levels and AS levels are generally obtained when leaving secondary school around 18 years of age; GCSEs and now-obsolete O levels and CSEs are generally obtained during secondary school around 16 years of age; NVQs, HNDs and HNCs are work-based, vocational qualifications. ^c^Counts do not sum to their corresponding totals because participants can select multiple answers.

### Variation in phenotypic disease profiles

**Fig. 3:**
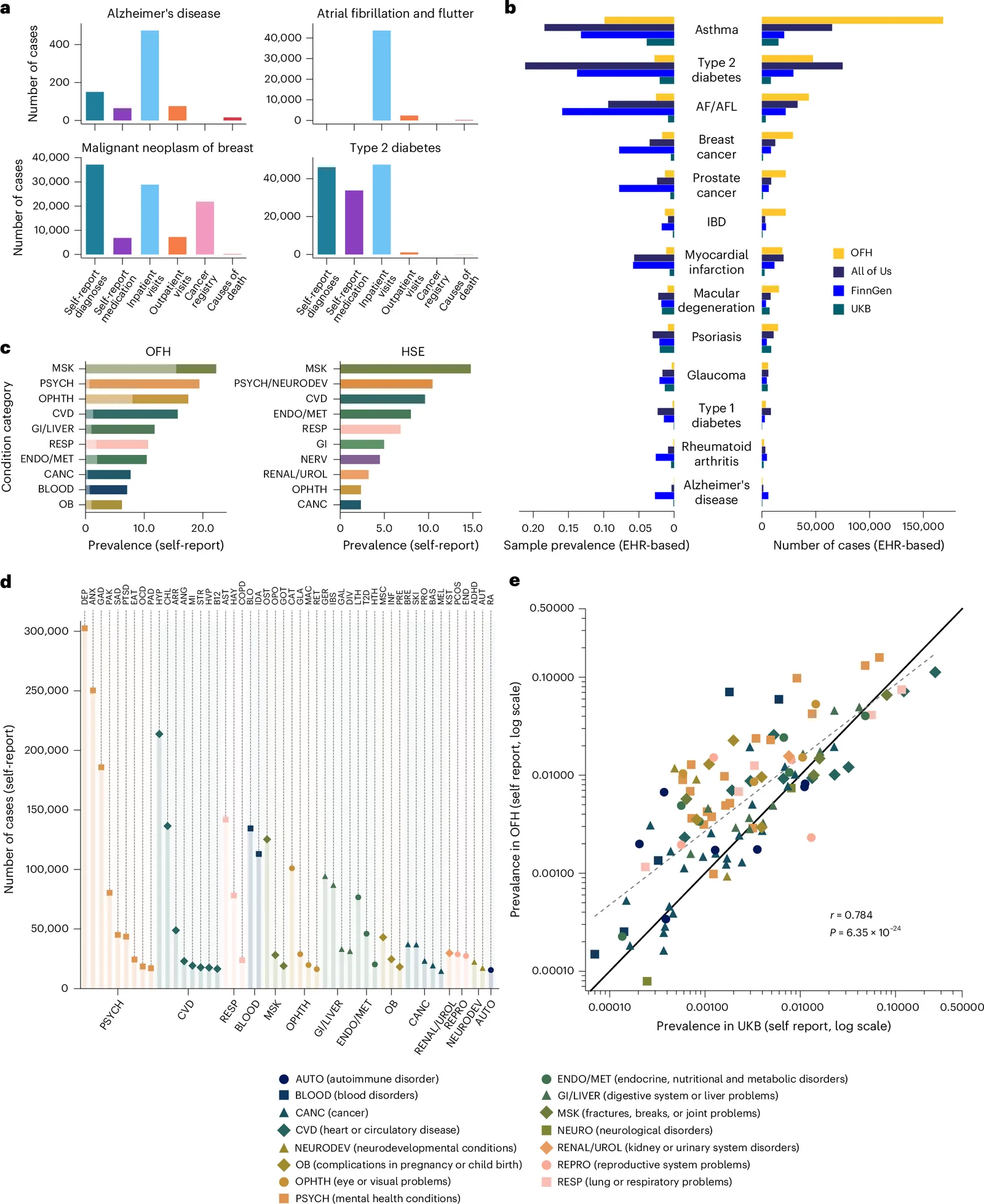
OFH disease phenotyping and comparison with other biobanks and UK population. **a**, Examples of data sources used for constructing four selected end points. Each individual can contribute only once to each data source, but the same individual can be counted in multiple data sources. **b**, The case prevalence and counts in OFH, All of Us, FinnGen and the UKB for several well-studied diseases (*N* = 13) sourced from hospital records (inpatient visits) using ICD-10 codes. The phenotypes are sorted on the basis of OFH prevalence. **c**, The prevalence of self-reported diagnoses for diseases in OFH compared with the HSE for 2022. Shaded areas represent cases that do not map onto specific diseases or conditions. **d**, The distribution of cases for self-reported diagnoses of common diseases and health conditions (*N* = 50), grouped by disease category. Each bar represents a disease or condition, with the *x* axis indicating the different disease category and the *y* axis representing the corresponding sample sizes. **e**, The case prevalence of self-reported diagnoses in OFH is compared with prevalence in the UKB. Each dot represents a specific condition, with the *x* axis showing the prevalence in UKB and the *y* axis showing the case proportion in OFH. The solid line indicates identity line, and the dashed line indicates the log–log linear regression. The correlation coefficient and significance were calculated using the two-sided Pearson correlation test. The bottom legend is shared across **c**, **d** and **e**. Definitions for specific disease abbreviations in **d** can be found in the Supplementary Tables. AF/AFL, atrial fibrillation/atrial flutter. Extended footnotes are provided in the Supplementary Information.

Across a wide set of phenotypes, we find that OFH provides large case counts and broadly concordant prevalence patterns compared with other major biobanks and national estimates (Fig. 3). Although the construction of end points for cases varies across self-report and linked electronic health records (EHRs; Fig. 3a and Supplementary Table 2), OFH expectedly has larger absolute case counts than several other large-scale national cohorts for many common conditions owing to its increased scale (such as, asthma, breast cancer and inflammatory bowel disease). In addition to the examples shown in Fig. 3a, we provide harmonized definitions and case counts for a further set of well-studied conditions in Supplementary Table 1. Using hospital-record-based phenotypes derived from International Classification of Disease Tenth Revision (ICD-10) codes (‘Phenotyping’ section in Methods) demonstrates the size of OFH compared with other large-scale national cohorts (‘Study populations’ in Methods). Focusing on 13 well-studied common diseases, we find that OFH has lower prevalence but yields substantially larger absolute case counts for most conditions compared with All of Us, FinnGen and the UKB (Fig. 3b). Notably, prevalence of type 2 diabetes is higher in the All of Us cohort than in OFH and the other European cohorts examined here, consistent with established differences in population prevalence between the USA and the UK^27,28^. Although differences in case definition, coding practice and healthcare systems probably affect absolute counts and must be considered when making cross-cohort comparisons, the absolute case count and number of controls (Supplementary Table 1) underscores the potential of OFH for clinical research. Comparisons of self-reported diagnoses between HSE and OFH participants highlighted additional systematic patterns (Fig. 3c,d), with several self-reported conditions more commonly reported in OFH than in estimates from the 2022 HSE, particularly mental health conditions and eye or visual problems (Fig. 3d and Supplementary Tables 5 and 6).

**Extended Data Fig. 3:**
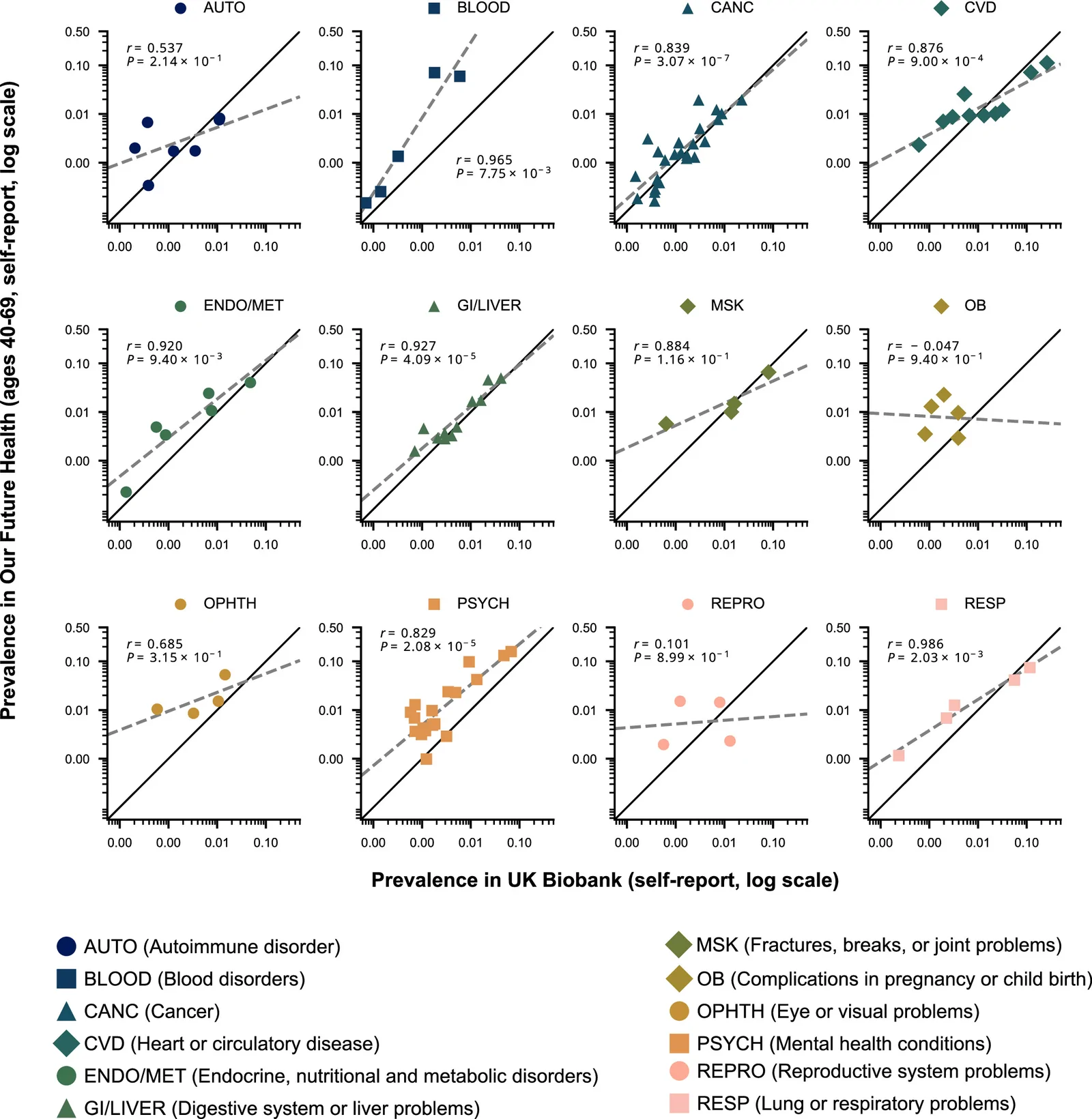
Comparison of prevalence estimates for self-reported health condition and disease diagnosis phenotypes by disease category in OFH and UKB. Significantly positive correlations are observed across self-reported for diagnoses across disease categories between prevalence in the UKB (x-axis) and OFH (y-axis), except reproductive system problems and complications in pregnancy or childbirth. Data for OFH participants are limited to participants aged 40–69 at the time of recruitment, in line with age composition of UKB participants. Disease categories are limited to those where the number of diseases or conditions >3. Correlation were assessed using a two-sided Pearson correlation test. Data are provided in Supplementary Table 6. OFH, Our Future Health; UKB, UK Biobank.

**Extended Data Fig. 4:**
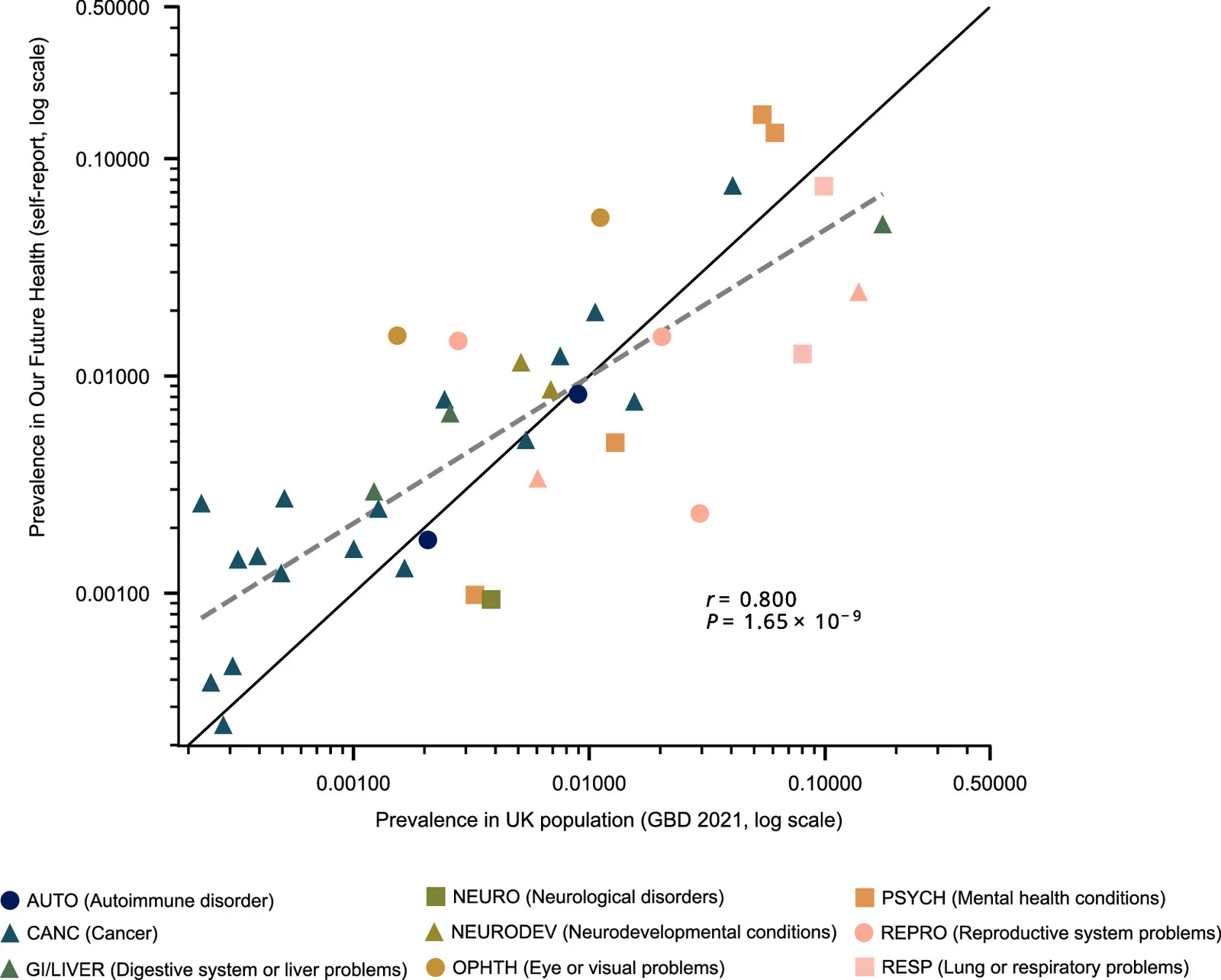
Prevalence estimates for self-reported health condition and disease diagnoses in OFH compared with national point prevalence estimates from the Global Burden of Disease Study 2021 for the United Kingdom. Each point represents a chronic health condition (*N*=38). The x-axis shows GBD point prevalence for each condition, and the y-axis shows the corresponding prevalence in OFH. The correlation was assessed using a two-sided Pearson correlation test (r = 0.80, p = 1.65 × 10^−9^). Data are provided in Supplementary Table 7. OFH, Our Future Health; GDB, Global Burden of Disease.

At the condition level, prevalence estimates for a set of common self-reported health conditions (*N* = 109) in OFH were strongly correlated with prevalence estimates from the UKB (Pearson *r* = 0.784, *P* = 6.35 × 10^−24^; Fig. 3e). Restricting analyses to participants aged 40–69 years yielded a similar correlation (*r* = 0.793, *P* = 1.01 × 10^−24^). Category-level analyses confirmed positive correlations for most disease classes, except for reproductive system conditions and complications related to pregnancy or childbirth (Extended Data Fig. 3), which may be related to differences in age structure (Fig. 1e). Despite the overall concordance, several other divergences were observed. Most notably, OFH participants reported substantially lower prevalence of age-associated cardiometabolic conditions, including hypertension, myocardial infarction and stroke, whereas depression, anxiety and related mental health conditions were more frequently reported than in UKB (Extended Data Fig. 3 and Supplementary Table 6). To further contextualize disease prevalence in OFH, we compared lifetime self-reported diagnoses with Global Burden of Disease (GBD) study for the UK (‘Study populations’ in Methods) point prevalence estimates for a restricted set of chronic, long-duration conditions (*N* = 38) (Extended Data Fig. 4). Despite differences in case definitions and ascertainment, the two sources showed a strong positive correlation (*r* = 0.80, *P* = 1.65 × 10^−9^), indicating broadly consistent relative ordering of common chronic conditions. However, absolute prevalence estimates should not be interpreted as directly comparable^21^, as OFH reflects lifetime diagnoses, whereas GBD estimates represent active disease at a specific time point (full condition-level comparisons are provided in Supplementary Table 7).

### Associations between health conditions and clinical correlates

**Fig. 4:**
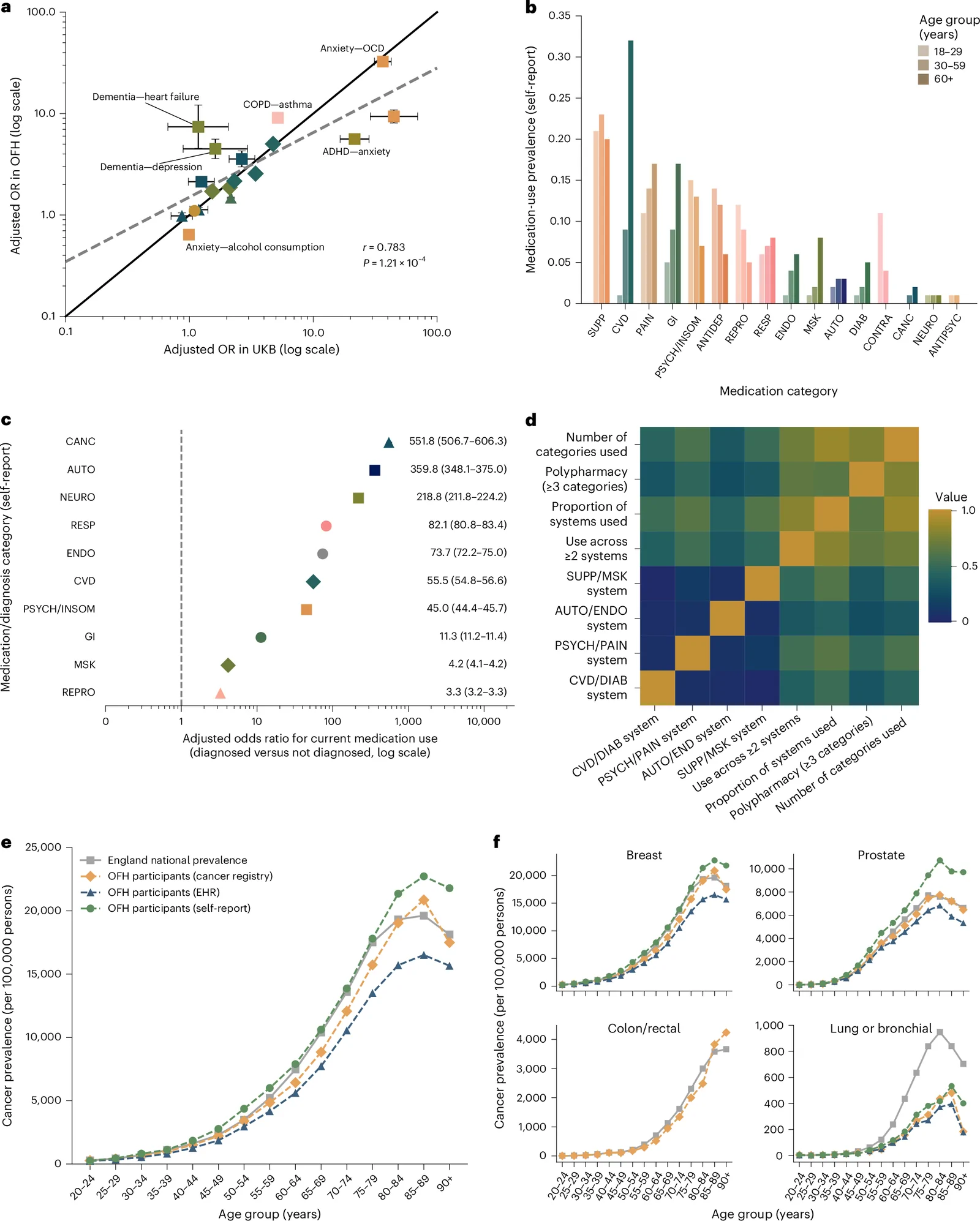
Clinical associations, medication use and cancer prevalence in OFH. **a**, Associations between several clinical correlate and health condition pairs (*N* = 18) in OFH and UKB. Each point represents the point estimate of an adjusted OR calculated via individual multivariable logistic regression models adjusted for age, sex, income and broad self-reported ethnicity. Error bars denote the 95% confidence intervals (CIs) around the estimate. The solid line indicates the identity line, and the dashed line indicates the log–log linear regression evaluated using a two-sided Pearson correlation test. **b**, The prevalence for current medication use across medication categories by age group. **c**, The associations between current medication use and corresponding diagnoses. Each point represents an adjusted OR (log scale). Error bars denote 95% CIs (shown in brackets). **d**, Pairwise correlations between medication-use summary phenotypes and system-level medication categories, computed using Pearson’s correlation coefficient for continuous–continuous pairs, point–biserial correlation for continuous–binary pairs, and the *φ* (phi) coefficient for binary–binary pairs. **e**,**f**, The age-specific prevalence of cancer diagnoses in OFH participants registered in England, with separate estimates by ascertainment source (self-report, linked cancer registry and linked inpatient EHRs), compared with national cancer prevalence estimates for England from the NDRS (rates per 100,000 persons): all cancers combined by age group (**e**) and selected cancer sites (**f**): breast (female), prostate (male), colon/rectal and lung or bronchial. Colors and abbreviations for **a**–**c** are defined as in Fig. 3d. Abbreviations for **b** are in Supplementary Tables.

Analyses of associations between selected health conditions and established clinical correlates, variables that relate to the underlying pathological or epidemiological process giving rise to particular disease or health outcomes, showed strong concordance with results from UKB (Fig. 4a and Supplementary Tables 3.1 and 3.2). Across *N* = 18 conditions, adjusted odds ratios (OR) estimated independently in the two cohorts were positively correlated (Pearson *r* = 0.783, *P* = 1.21 × 10^−4^), with similar effect sizes observed when restricting OFH participants to the age range 40–69 years (*r* = 0.767, P = 2.05 × 10^−4^). Well-established associations, including those between cardiovascular disease and hypertension, respiratory disease and smoking, metabolic conditions and adiposity, psychiatric conditions and family history and cancer outcomes and smoking or family history, were consistently replicated in OFH, supporting the validity of phenotyping and covariate capture in this cohort. Although effect-size estimates were broadly aligned across cohorts, several differences in magnitude were observed, probably reflecting variation in cohort composition, disease prevalence and ascertainment methods. Stratified analyses by self-reported ethnicity indicated that several associations varied in strength across population groups within OFH, while remaining directionally consistent with UKB (Supplementary Table 3.2 and Supplementary Information Section 2.3).

### Medication-use patterns, correlation with diagnosis and cross-source concordance of self-report

Medication use showed clear age-related patterning, with supplements and pain-relief medications commonly reported across all age groups, and increasing use of heart and circulatory, bone health and cancer therapies with older ages (Fig. 4b and Supplementary Table 8). Multivariate analyses linking current medication use to corresponding self-reported diagnoses yielded large, disease-specific ORs, including for cancer (OR ~550), autoimmune disorders (OR ~360) and neurological disorders (OR ~220), indicating strong concordance between reported diagnoses and medication use (Fig. 4c and Supplementary Table 9.1). Stratified analyses by self-reported ethnicity showed that these associations were directionally consistent across groups, although effect sizes varied in magnitude for several medication–diagnosis pairs (Supplementary Table 9.2).

**Extended Data Fig. 5:**
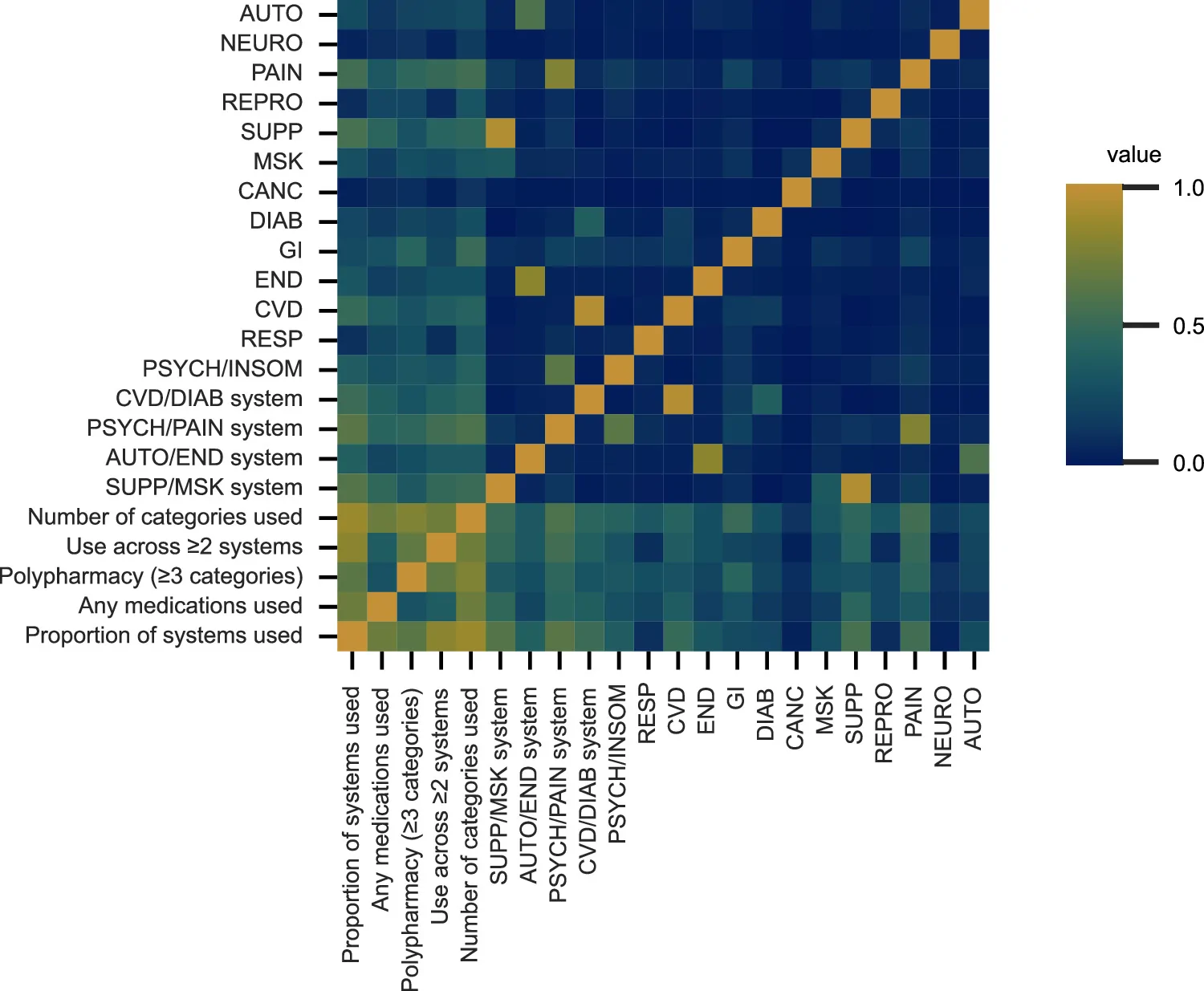
Correlation metrics between the medication use pattern phenotypes. Correlation between each phenotype combination calculated in the overlapping sample intersection (Pearson’s correlation coefficient for continuous-continuous phenotype pairs, Point-biserial correlation coefficient for continuous-binary pairs, and Phi-coefficient for binary-binary pairs). Abbreviations are provided in Supplementary Table 1 and data are provided in Supplementary Table 12.

**Extended Data Fig. 6:**
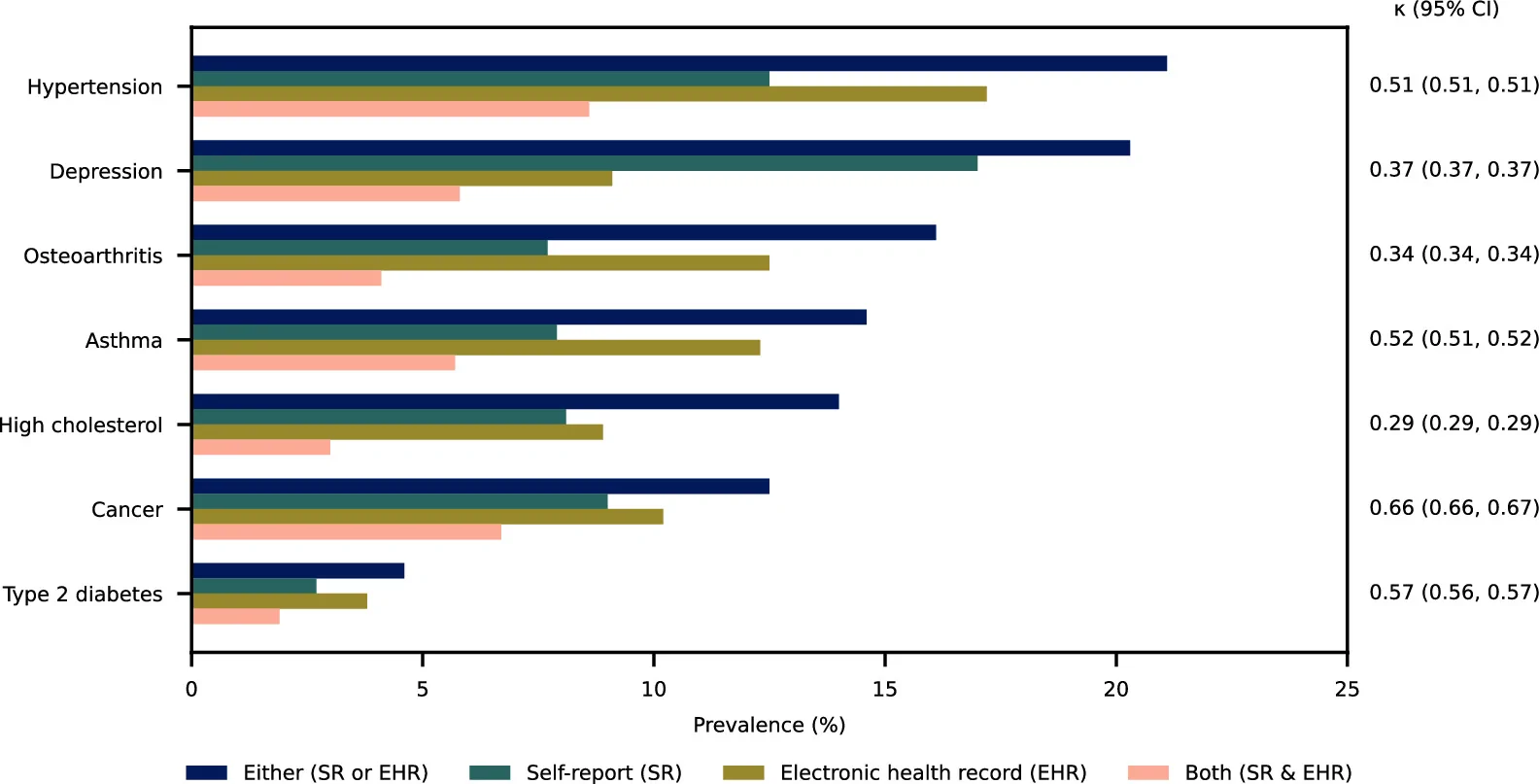
Prevalence of selected chronic conditions by data source and agreement between self-report and electronic health records in Our Future Health. Correlation between each phenotype combination calculated in the overlapping sample intersection (Pearson’s correlation coefficient for continuous-continuous phenotype pairs, Point-biserial correlation coefficient for continuous-binary pairs, and Phi-coefficient for binary-binary pairs). Abbreviations are provided in Supplementary Table 1 and data are provided in Supplementary Table 12.

Correlation analyses of medication-use phenotypes revealed coherent patterns of co-use, including clustering of mental health and pain-relief medications and of supplements with bone-health medications (Fig. 4d and Extended Data Fig. 5). Summary measures of medication use, including the number of medication categories, polypharmacy, proportion of systems used and multisystem use, were strongly correlated (*r* ≈ 0.7–0.8), reflecting overlapping aspects of medication-use behavior (full details provided in Supplementary Table 13). Alongside concordance between self-reported diagnoses and medication use, additional analysis comparing self-reported diagnoses with linked inpatient EHR-derived diagnoses for several common chronic diseases showed that agreement varied by condition (Extended Data Fig. 6 and Supplementary Table 15). Cohen’s *κ* ranged from 0.29 for high cholesterol to 0.66 for cancer. Concordance was generally higher for clinically salient and well-defined conditions, including cancer, whereas lower agreement was observed for conditions more susceptible to differences in ascertainment and healthcare setting, including high cholesterol and depression.

### Cancer prevalence in comparison with national estimates

Age- and sex-specific prevalence of cancer diagnoses among OFH participants registered in England was assessed using self-reported diagnoses, inpatient EHR records and linked cancer registry data and compared against national estimates from the National Disease Registration Service (NDRS) (Fig. 4e,f and Supplementary Table 12). For all cancers combined, prevalence estimates across the three OFH data sources were broadly comparable to national figures at younger ages, with self-reported prevalence tending to exceed both EHR- and registry-derived estimates at older ages; for example, among persons aged 85–89 years, the self-report rate was 22,728 per 100,000 compared with 16,514 per 100,000 from inpatient EHR and 20,861 per 100,000 from cancer registry data, against a national estimate of 19,622 per 100,000.

Breast cancer prevalence followed a similar pattern, with self-reported rates in OFH exceeding national estimates across most age groups from midlife onwards, whereas EHR- and cancer registry-derived estimates were more closely aligned with or modestly below NDRS figures. Prostate cancer prevalence among male participants showed a broadly analogous pattern, with self-reported estimates at older ages exceeding both national figures and EHR-derived estimates. Colon and rectal cancer prevalence was broadly comparable across all three OFH data sources and tracked national estimates closely across most age groups in both sexes. In contrast, lung and bronchial cancer prevalence was substantially lower in OFH than in national estimates across all three data sources, in both female and male participants and at all ages with sufficient counts. The convergence of this underrepresentation across independent ascertainment methods indicates that the deficit in lung cancer burden within OFH probably reflects true differences in disease prevalence, consistent with potential differences in risk factor profiles within the cohort (Tables 1 and 2). Together, these findings indicate that differences in overall cancer prevalence between OFH and national estimates vary by data source and cancer site, with inflation apparent in self-reported prevalence for breast and prostate cancer at older ages, potentially indicating recall bias (box 1 in the Supplementary Information) and sample ascertainment differences whereby self-reported diagnoses may capture borderline or preinvasive lesions and a robust and consistent underrepresentation of lung cancer across all ascertainment methods consistent with the cohort’s risk factor profile.

### Prevalence of less common conditions and rare diseases

**Extended Data Fig. 7:**
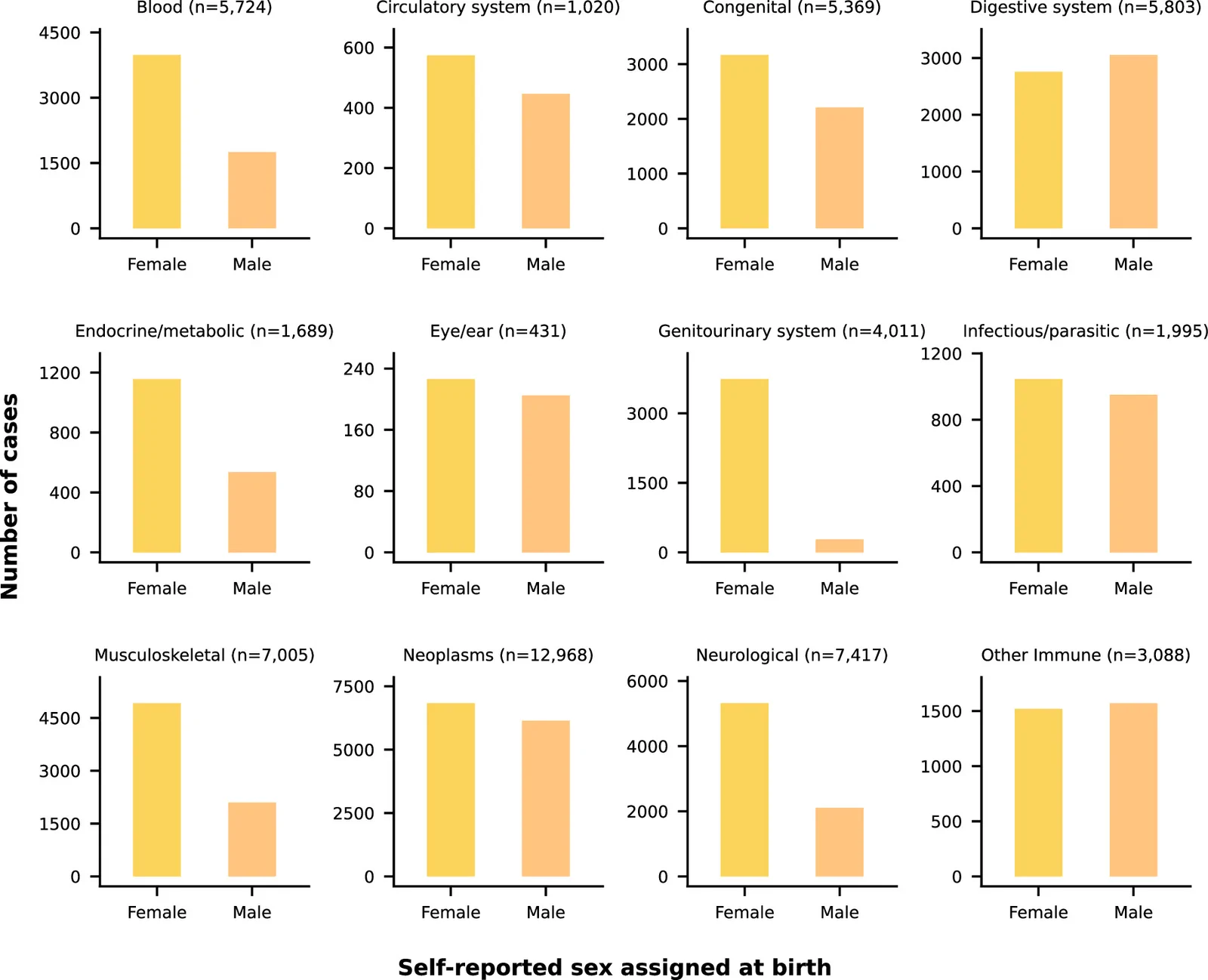
Case counts for different groups of rare conditions in Our Future Health by self-reported sex assigned at birth. Each bar plot presents the number of male and female individuals in Our Future Health who have at least one rare condition from a particular group from a list of rare conditions (*N*=187). Non-overlapping groups of rare conditions were identified from their corresponding ICD-10 codes. For a description of the selection of rare conditions see Methods and Supplementary Information. List of rare conditions and ICD-10 codes are provided in Supplementary Table 16.

Although our focus is on common conditions and phenotypes relevant to public health, it is also important to consider the potential of increased numbers of OFH participants with less common, understudied conditions, including rare diseases (defined in Europe as those affecting fewer than 1 in 2,000 people)^29^. In an exploratory analysis using a set of less common conditions (*N* = 187; Supplementary Table 16), including rare diseases meeting the European rarity prevalence threshold and selected low-prevalence conditions previously identified in the UKB^30^, OFH participants exhibit considerable differences in sex proportion between conditions. For instance, rare digestive system conditions are more frequent among male participants, whereas blood, endocrine and metabolic, and musculoskeletal conditions are more frequent among female participants (Extended Data Fig. 7). Several conditions exhibited marked sex imbalance, including systemic lupus erythematosus (*N* = 1,825*;* 90.4% female) and interstitial cystitis (*N* = 1,409; 91.0% female) consistent with known sex-specific disease biology^31^ and female-to-male ratios found in studies of rare disease prevalence in the UK^32^.

**Extended Data Fig. 8:**
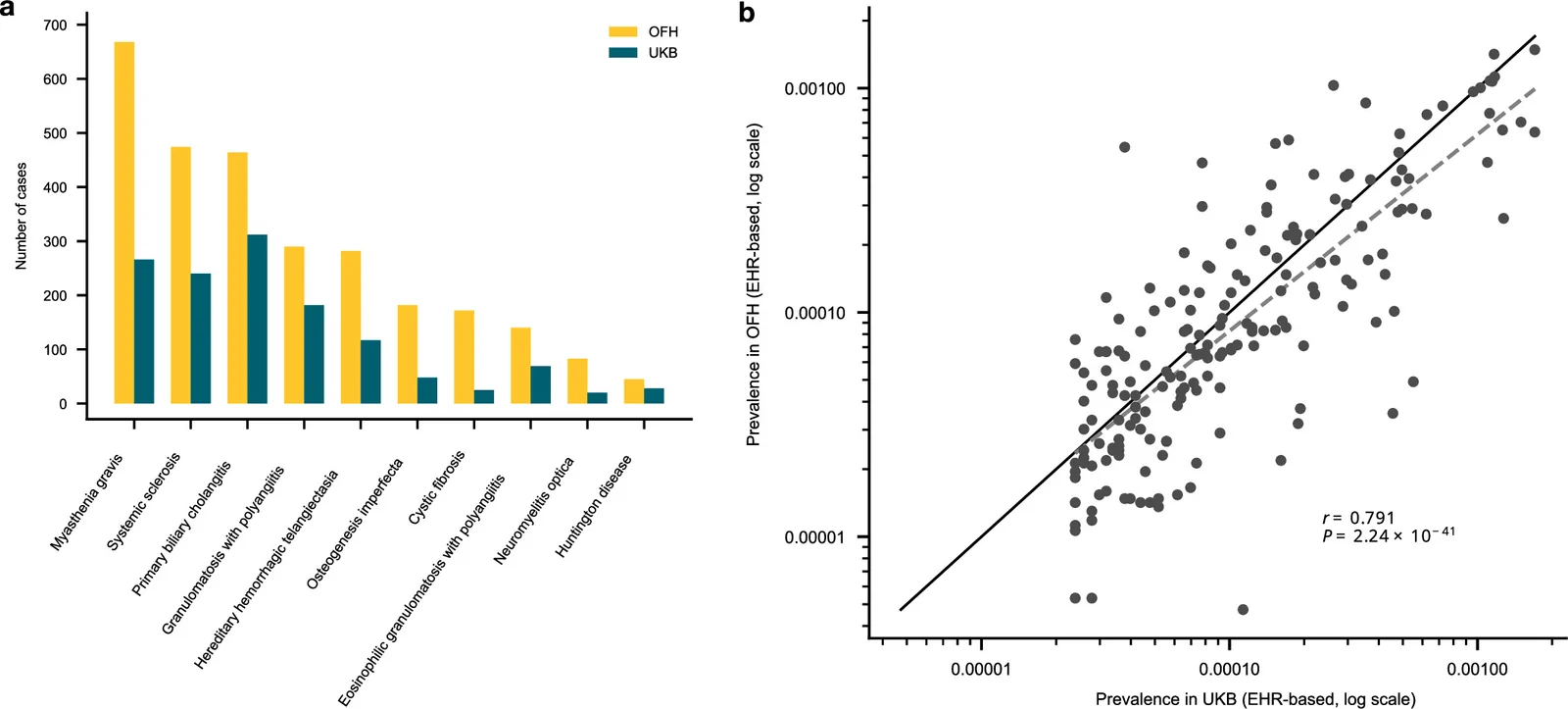
Representation of rare conditions in Our Future Health and UK Biobank. **a**, Case counts for selected rare health conditions (*N*=187) in OFH and UKB. Bars show the number of individuals diagnosed with each condition in each cohort (see Methods); ten exemplar conditions spanning immune-mediated, genetic, and neurological disorders are shown. **b**, Scatter plot comparing prevalence estimates of rare conditions in OFH and UKB. Each point represents a condition. Solid line indicates identity line, and the dashed line indicates the log–log linear regression. The correlation coefficient and significance were calculated using the two-sided Pearson correlation test. Despite larger absolute case counts in OFH, prevalence estimates are correlated between cohorts. List of rare conditions and ICD-10 codes are provided in Supplementary Table 16. OFH, Our Future Health; UKB, UK Biobank.

In terms of specific rare disease case counts, OFH consistently provides substantially greater cases than UKB (Extended Data Fig. 8a and Supplementary Table 16); for example, OFH includes 668 individuals with myasthenia gravis (versus 266 in UKB), 464 with primary biliary cholangitis (versus 312), 172 with cystic fibrosis (versus 25) and 83 with neuromyelitis optica (versus 20). Despite these larger absolute counts, prevalence estimates for the whole set are broadly correlated between cohorts (Extended Data Fig. 8b; *r* = 0.79, *P* = 2.24 × 10^−^^41^), although several conditions showed markedly disproportionate representation beyond what cohort size alone would predict, including idiopathic intracranial hypertension (1,739 versus 132 cases in UKB) and complex regional pain syndrome type 2 (922 versus 19), probably reflecting OFH’s younger age profile and broader recruitment criteria. Conversely, pleural mesothelioma, which is strongly associated with older age, was underrepresented relative to UKB (83 versus 277). These divergences probably reflect a combination of cohort age structure, survivorship, healthcare utilization and differences in recruitment period between OFH and UKB. Further work is required to systematically assess these mechanisms and comprehensively evaluate representativeness of rare conditions in OFH relative to recent UK nationwide estimates^32,33^.

## Discussion

OFH has a large scale, scope and potential for biomedical and clinical research but this potential needs to be seen within the context of the currently available data and the limitations they bring. With baseline data on approximately 2 million registered participants, it already constitutes the largest consented cohort of its kind^22^ (Fig. 3). This is particularly relevant for underserved populations, stratified analyses and potentially rare conditions. A related key feature of OFH is its capacity for multiple, complementary phenotyping across data sources (Fig. 1b). This will in time be strengthened through repeated follow-up questionnaires (for example, cognitive function, diet and pain)^23^, revealing time-varying exposures and regularly updated linkage to national health records, enabling prospective analyses of incident outcomes, as outlined in the OFH protocol. In parallel, the active clinical research recruitment service (https://research.ourfuturehealth.org.uk/clinical-research-recruitment/) enables prevention and early detection trials and studies of selected subcohorts (Extended Data Fig. 1).

As OFH is ongoing, a final response rate cannot yet be produced, although OFH has estimated a rate of 4.5% on the basis of the proportion of participants who have consented from the participants that have been invited to take part^23^. Similar to suggestions made by other cohorts such as the UKB^15^, OFH should not currently be used for deriving generalizable disease prevalence and incidence rates across all conditions. This limitation is particularly relevant for rare conditions, as approximately 70% of rare diseases begin in childhood, and nearly one third of affected patients die before the age of 5 years^33^, meaning many rare conditions are inherently underrepresented in an adult volunteer cohort such as OFH, and representativeness itself remains difficult to establish for severe early onset diseases and conditions affected by survivorship. Nevertheless, the scale of OFH and its linked phenotypic and genomic data can still support methodological advances in rare disease research, including improved rare disease phenotyping, risk stratification, multimodal analyses and recruitment into future studies.

Crucially, the composition of OFH needs to also be considered in relation to contextual factors that influence ascertainment, with implications for downstream analyses. Most notably, the comparatively high burden of self-reported mental health conditions (Fig. 3d) may partially reflect changes in self-reporting and subsequent diagnoses of mental health conditions over time^34^, as well as the differential and persistent impacts of UK austerity measures^35^ and global shocks including the 2020–2022 coronavirus disease 2019 (COVID-19) pandemic^36^. The effects of COVID-19 and the post-pandemic period on UK population health have been lasting and heterogeneous, with variation observed across physical disease^37^, mental health^38^ and health behaviors^39^. The higher level of male participants reporting frequent drinking in OFH participants, registered between May 2021 and June 2024, compared with 2021 HSE estimates, aligns with research on sustained elevated levels of alcohol consumption relative to prepandemic levels^40^. In a robustness check, we examined whether prevalence estimates for health behaviors, mental health diagnoses and medication use differed between earlier and later recruitment waves and found estimates to be broadly stable over time (Supplementary Table 14). However, as all recruitment occurred after the acute phase of the COVID-19 pandemic, further comparison with external datasets containing pre- and peripandemic measures can help assess potential COVID-19-related cohort effects. Similarly, contemporaneous pressures, such as prolonged National Health Service (NHS) waiting times^41^, may also have selectively attracted participants with unmet healthcare needs to participate^42^.

Going forward, researchers using OFH for causal inference first need to consider the extent to which their study might be affected by selective participation and collider bias^43,44^(box 1 in the Supplementary Information). Although we report some participation bias in OFH, studies have demonstrated that nonrepresentativeness does not necessarily lead to substantial bias in exposure–disease associations^45^. One way for future studies to empirically demonstrate this is to replicate and benchmark results against other similar populations. This can be leveraged by comparing across other large cohorts and potential trans-biobank research^21^. Given its size, the OFH sample is also expected to include many pairs of participating genotyped first-degree relatives. This allows using the available genetic data (Fig. 1b) to infer kinship networks among OFH participants, enabling future family-based and within-household analyses that can help clarify causal relationships between putative risk factors and outcomes^46^. The presence of first-degree relatives also facilitates the study of the genetic basis of participation bias (box 1 in the Supplementary Information) using only the genotypes of the participants^47^, which in turn can be used to determine whether sample weighting based on phenotypes alone could effectively adjust for participation bias in analyses^48^.

One key limitation of our study is that we rely primarily on self-reported participant data, which may incompletely capture chronic and ambulatory conditions relative to primary care EHR data, as previously demonstrated in UKB^49^; given primary care EHR data is not yet included, current EHR-derived definitions should not be treated as a definitive gold standard. In terms of demographic and sex-differential participation, OFH participants were more likely to report being female across all major ethnic backgrounds (Extended Data Fig. 2). Male and female participants were less likely to report being current smokers compared with national HSE estimates but more likely to report frequent alcohol consumption, particularly among male participants. However, rates of lung or bronchial cancer were lower in OFH participants (Fig. 4d) across self-report and linked EHRs. These findings are not wholly consistent with the more uniform ‘healthy volunteer’ bias found in other cohorts^7,15^ and may underscore the more diverse sampling frame or be plausible consequences of differential screening uptake and smoking prevalence within the cohort (Table 1).

OFH provides a growing resource for biomedical research, evidenced by emerging findings^50^, yet it is important to also acknowledge the limitations of currently available data. As recruitment continues and additional EHR data become available (Fig. 1a), the continuous evaluation of cohort composition will be important^21^, along with the investigation of potential recall bias and nonrandom-missingness of item-level nonresponse behaviors. To enable more reproducible analyses, we provide an open-source Python library, Phenofhy^51^, for researchers using OFH phenotype data. The longer-term success of the program will depend on additional data linkage and its use in real-world clinical settings^52^, additional investigation of the utility of data that may be given to participants^23^ and continuous collaboration with participants to understand whether they trust how their data are used^42^, reflective of the broader challenges faced by the UK health data community^53^. Taken together, OFH is already a valuable resource, but ensuring that the program realizes its potential and benefits all must remain a shared priority.

## Methods

### Ethical approval

This study involves data from human participants that have participated in the OFH program and the UKB. Ethical approval for OFH was granted by the Cambridge East Research Ethics Committee (REC reference: 21/EE/0016). The UKB has approval from the North West Multi-Centre Research Ethics Committee as a Research Tissue Bank. All participants provided written informed consent before participation in either study.

### Study populations

OFH is a nonprobability, volunteer-based prospective cohort. The sampling frame comprises the UK-resident adult population (aged ≥18 years). Participants were recruited via in-person appointments at centers and pop-up clinic locations across England, Wales and Scotland and through invitation letters sent to all individuals and households, typically within a 5-mile radius of a newly opened center^23^ (Fig. 1c; recruitment in Northern Ireland only begun in late 2025), drawn from more than 2.5 million participants who have consented to take part (Extended Data Table 2). The recruitment approach first prioritized the inclusion of participants from more deprived areas and from UK minoritized ethnic groups that have been historically underrepresented in health research (Fig. 1), as recently described^23^. Our analyses included approximately 1.9 million participants (total sample size varies slightly across analyses owing to variation in samples sizes across data sources and differing exclusion criteria). Available data from consented participants included responses from the baseline questionnaires (participants completed either version 1 or version 2), which comprised up to 288 questions across five sections (Fig. 1b), along with clinical measurements, geolocation data and linked EHRs. OFH releases new data on a quarterly basis; we relied on OFH data from releases 12 and 13, released on 17 September and 11 December 2025, respectively. Information on the latest data release is available at https://ourfuturehealth.gitbook.io/our-future-health/data-releases/current-data-release.

In our study, analyses were restricted to participants with a valid age at registration (≥18 years). For analyses including ethnicity, participants who self-reported ‘do not know’, ‘prefer not to answer’ or did not provide a response were excluded. Analyses of questionnaire items unique to version 2 (‘Phenotyping’ section) were restricted to participants who completed either v2.1 or v2.2 of the questionnaire as part of data release 12 (97.2%; *N* = 1,847,750), unless otherwise indicated. Percentages are at times reported relative to the full cohort for consistency with cohort-wide reporting; therefore, totals may not always sum to 100%. Throughout, prevalence is defined as the proportion of OFH participants reporting or diagnosed with a particular condition, ascertained from the available data sources used in each analysis (for example, self-report or linked health records; ‘Phenotyping’ section). Unless otherwise specified, age refers to self-reported age at registration; sex refers to self-reported sex assigned at birth; and ethnicity refers to self-reported ethnicity using the list of Level 2 ethnic groups defined by the UK Government and adapted by OFH.

To situate the prevalence of health conditions and diseases in OFH within the context of other international cohorts, we also compared prevalence for multiple well-studied common diseases (*N* = 13) to three other national, large-scale biobanks for which we had access to comparable data: All of Us^4^, FinnGen^6^ and the UKB^3^. Given the UK-focus of OFH, and the fact that the baseline questionnaire was intended to align closely with the UKB (https://ourfuturehealth.org.uk/our-research-mission/how-our-future-health-works/), we conducted further cross-cohort comparisons between both cohorts. The UKB is a prospective, population-based study of half a million participants aged 40–69 years recruited across the UK between 2006 and 2010. The cohort design, recruitment procedures and data collection methods are described elsewhere^3^. Although the UKB has been found to not be suitable for deriving generalizable disease prevalence^15^, its large size and deep phenotyping have made it a valuable resource for public health research^2^; hence, we focus on it here. To assess comparability between cohorts, we performed sensitivity analyses matching the OFH and UKB cohorts on key demographic factors (restricted to participants aged 40–69 years who self-reported sex assigned at birth as male or female) (Extended Data Fig. 3 and Supplementary Tables). Notably, as UKB and OFH participants of equivalent age belong to different birth cohorts, several observed divergences in condition prevalence may still reflect birth cohort effects relating to generational shifts in exposure or disease risk.

To compare OFH with the general UK population, we selected nationally representative survey data that best matched the demographic composition (sex, age and country of registration) and initial recruitment period of OFH. When characteristics in national survey summaries were reported only in aggregated age, sex subgroups or only for specific countries of the UK, OFH participants were stratified or restricted to corresponding categories for comparability. Following ref. ^23^, our comparisons focus primarily on England, Wales and Scotland, where OFH recruitment invitation and in-person appointments started (appointments in England began in late 2022 and appointments in Wales and Scotland began in early 2024). Recruitment in Northern Ireland only begun in late 2025, and hence, data from participants in this country are not included in data releases 13 or 12 used in this study; the small percentage of participants who self-reported living in Northern Ireland (0.2%) at the time of registration probably represent participants who traveled to England, Scotland or Wales for a clinic measurements appointment.

Data on ethnicity were obtained from the 2021 census for England and Wales^55^ and the 2022 census for Scotland^56^^,57^, referred to throughout as the 2021–2022 UK census for simplicity. Anthropometric, lifestyle and health-related characteristics were obtained from the HSE for the years 2021 and 2022 (ref. ^54^). The HSE is an annual cross-sectional survey of a representative sample of private households in England, selected through a two-stage random probability design, which collects data on health conditions and health-related behaviors. Data on disease prevalence in the general UK population were obtained from the most recent GBD study at the time of writing^58^. The methodology for GBD estimates has been described in detail elsewhere^59^. Analyses were restricted to the year 2021. Data on national cancer prevalence were obtained from the NDRS^60^, which provides counts and rates of individuals living in England with or beyond a cancer diagnosis as of 31 December 2022 (for diagnoses since 1 January 1995).

Although not a focus herein, OFH also already provides a growing genetic and biological resource^21^, blood samples for genotyping on a custom 700,000-variant array, with approximately 750,000 participants with genotyped and imputed genotype array data. Other samples will in time enable additional omics assays (for example, proteomics, whole-genome sequencing (WGS) and methylation)^23^. OFH therefore already provides a complementary resource to existing resources through its combination of very large-scale active recruitment, data linkage and infrastructure for translational research. However, its relative utility compared with established large-scale cohorts such as All of Us, China Kadoorie Biobank, Million Veteran Program and UKB will still depend on the specific research question, study design and data modalities required (see Supplementary Table 13 for a brief overview of these biobanks compared with OFH).

### Phenotyping

Owing to the timing of our study and only a marginal increase in baseline phenotypic data between data releases (1.5%), we relied on data release 12 for most primary analyses, which included: (1) self-reported data from participants who submitted participant demographic data and either version 1 (*N* = 52,745) or version 2 (*N* = 1,847,812) of the baseline questionnaire (version 2 includes additional questions and allows participants to respond to new or updated questions; further details and OFH’s versioning approach are described at https://ourfuturehealth.gitbook.io/our-future-health/data/questionnaire-data) on or before 15 July 2025 (total *N* = 1,900,557); (2) clinic measurements data from participants who attended an appointment on or before 1 April 2025 (*N* = 1,433,275); and (3) linked EHRs (including inpatient visits, outpatient visits, cancer registry and death registration data available for participants registered in England) for participants who completed a questionnaire before 9 April 2025 and were successfully linked to a NHS number (*N* = 1,703,250), with *N* = 1,665,668 having at least one linked secondary care or death registration record. These records span multiple EHR sources and time windows; therefore, case ascertainment and follow-up vary by dataset. We relied on data release 13 to analyze the geographic distribution of OFH participants (*N* = 1,841,458), as it included significantly more self-reported country and regional location data compared with data release 12 (*N* = 102,103) and the prevalence of selected uncommon and rare conditions (*N* = 187) in participants with linked EHRs (*N* = 1,690,845). To maximize sample size, all available data were included where possible; consequently, total sample size varied across analyses.

To characterize the OFH cohort, we used participant self-reported data on sociodemographic indicators, region of registration, lifestyle exposures, family health history and personal health history including past diagnoses and current medication use, obtained from the baseline questionnaire. Data on height, weight and waist circumference were obtained from the clinic measurements data. Exact details of the questionnaire and how the clinic measurements are taken are described elsewhere^23^. To provide examples of how disease end points can be constructed from different OFH datasets and explore prevalence of rare conditions, we further used data from EHRs, specifically, inpatient visits (admitted patient care episodes at NHS hospitals in England) and outpatient visits (outpatient appointments at NHS hospitals in England) collected by NHS England and linked to OFH. We also used linked cancer registry (patient tumor-level) data from the NDRS and linked death registration (cause of death) mortality data collected by the Office for National Statistics (ONS). Cancer prevalence was calculated in OFH for all cancers combined (excluding nonmelanoma skin cancer) and common types (breast, prostate, colon/rectal and lung or bronchial cancer) using self-report and EHRs, comprising both inpatient visits and linked cancer registry patient tumor data. Diagnostic information in linked EHR datasets was encoded using the ICD-10, and case ascertainment included primary and secondary diagnoses. For cancer analyses, we restricted case ascertainment to primary diagnoses recorded in linked cancer registry and inpatient datasets. Full details of all OFH data that has been measured and linked is available on the dedicated researcher website (https://research.ourfuturehealth.org.uk/data-and-cohort/).

We additionally derived several phenotypes using the Python library Phenofhy^51^, created as a companion to this paper and for other OFH users, including: age at registration, smoking status, BMI, physical walking activity (walking ≥16 times a month for at least 10 min) and multiple medication-use variables (number of medication categories used, polypharmacy, proportion of medication systems used and use across ≥2 systems). Where possible, we also sought to harmonize categorical variables to be consistent with UKB reporting^15^ for ease of comparison (see ‘Derived phenotypes’ section for details of the derivation logic).

To compare case prevalence for (*N* = 13) well-studied diseases between OFH, All of Us, FinnGen and the UKB, phenotypes were defined using ICD-10 codes in linked EHR. Data for FinnGen and the UKB were obtained from prior work^6^, and data for All of Us were obtained from the All of Us data snapshots. For cross-cohort prevalence comparisons, harmonized ICD-10 phenotype definitions were applied consistently across datasets where possible, with case definitions based on the presence of at least one qualifying ICD-10 diagnosis code. To robustly compare prevalence of diseases and health conditions between OFH and the UKB, cases were identified using participant self-reported diagnoses in both cohorts. For each self-reported disease or health condition in OFH, we reviewed each possible match in UKB non-cancer illness codes or ever diagnosed mental health conditions and linked it to the equivalent self-reported condition. A total of *N* = 109 shared phenotypes were identified. In the absence of standardized mappings (see ref. ^21^ for a discussion), to compare prevalence between OFH and the UK population using the GBD, we mapped self-reported health conditions to GBD level 3 and 4 causes that were clinically equivalent and rejected any mapping where aggregation would distort comparisons. This conservative approach resulted in a total of *N* = 45 shared phenotypes.

Finally, to provide an initial, exploratory assessment of case numbers for less common and rare conditions in OFH participants, we analyzed linked inpatient EHRs using the ICD-10-to-ORPHA code^61^ (an extensive online resource for rare diseases) mappings adapted from the consensus framework approach used in prior work examining rare diseases in the UKB. This involved using mappings in which the selected ORPHA code is as specific as possible, but no more specific than the corresponding ICD-10 code, thereby enabling reliable identification of rare conditions from ICD-10-coded diagnoses (see section 2.16 in the Supplementary Information for further details). In OFH, we analyzed *N* = 187 conditions previously characterized in UKB^30^ (Supplementary Table 16), including clinically defined rare and ultra-rare diseases as well as selected low-prevalence conditions retained for biobank comparison purposes. Cases were defined as participants with at least one qualifying ICD-10 code recorded in either primary or secondary inpatient diagnoses corresponding to the mapped ORPHA definitions. Prevalence was estimated by dividing the number of individuals with mapped ICD-10-coded diagnoses by the total number of OFH participants with linked inpatient EHR available. Further details of all mapping processes, including limitations, are discussed in the Supplementary Information, and the full list of mapped phenotypes are provided in the Supplementary Tables.

### Derived phenotypes

Age at registration was derived from self-reported date of birth and date of registration. BMI was calculated as weight (kg) divided by height squared (m^2^), using clinic measurements (Supplementary Tables 4.1 and 4.2). Self-reported alcohol-consumption categories were harmonized to UKB conventions^15^ to support cross-cohort comparison (Table 1 and Supplementary Tables 4.1 and 4.2).

Smoking status was derived using questionnaire-specific logic. Current smokers reported cigarette smoking at any frequency. Never smokers reported no current smoking and fewer than 100 lifetime smoking occasions (including non-cigarette products). Former smokers reported no current smoking but either daily or most-days smoking previously or at least 100 lifetime occasions. Questionnaire-version-specific variables are listed in Supplementary Tables 4.1 and 4.2 footnotes.

Physical activity was defined as walking at least 16 times per month for at least 10 min, derived from questionnaire version 2. Medication-use indicators and aggregated medication-pattern variables were derived using the Phenofhy package and are summarized in Supplementary Tables 8, 9 and 12.

### Standard exclusions

Participants with invalid age at registration (coded −999) were excluded from age-adjusted analyses (all regression models; Supplementary Tables 3 and 9). Sex-specific analyses excluded participants reporting intersex, ‘prefer not to answer’, or missing sex. Analyses involving ethnicity excluded ‘do not know’, ‘prefer not to answer’ and missing responses.

Variables introduced only in questionnaire version 2 were analyzed among questionnaire version 2 respondents (Supplementary Tables 4–9). For cohort-wide descriptive reporting (Table 1 and Supplementary Tables 4 and 5), several percentages are presented relative to the full cohort; version-restricted denominators are noted explicitly in table footnotes.

Alongside standard exclusions for quality control, we also applied additional exclusions to comply with the OFH Statistical Disclosure Control (SDC) framework, which aims to minimize the risk of participant re-identification. In line with the SDC data guidelines (https://dnanexus.gitbook.io/ofh/airlock/safe-outputs-data-guidelines-for-export), throughout our Results and Supplementary Tables reporting, we do not report extreme values (for example, minimum and maximum values) or exclude frequencies or counts relating to all individuals in the study population or all but one individual, and we replaced cell values with ‘<10’ for counts or frequencies based on a small group (<10 observations).

To further minimize the risk of participant re-identification risk (disclosure), all research using the OFH TRE additionally follows the four-eyes principle, meaning that every output was scrutinized by two people at a minimum, including at least one person not involved in the research.

### Statistical analysis

We summarized the sociodemographic, lifestyle and health-related characteristics of OFH participants and compared these distributions with estimates from the nationally representative HSE 2021/2022. Continuous variables are reported as mean ± s.d. and categorical variables as counts and proportions; percentages were calculated relative to the number of participants with available data for each variable. Self-reported regional representation was compared with UK census estimates from 2021 to 2022 (Fig. 2a,b). Regional representation was visualized using a log_2_ representation index, defined as log_2_(OFH proportion/UK population proportion), displayed on a symmetric scale.

To assess correspondence in disease prevalence between OFH and the UKB, we compared the prevalence of shared self-reported diseases or health conditions (Fig. 2e, Extended Data Fig. 3 and Supplementary Table 6), as well as for rare conditions defined using the consensus ICD-10-to-ORPHA mappings we use (Extended Data Fig. 8 and Supplementary Table 16). Prevalence was plotted on a log–log scale after exclusion of missing values. The Pearson correlation coefficient (*r*) was calculated between log_10_-transformed prevalence estimates for OFH and UKB, and a log–log linear regression was fitted to characterize the overall multiplicative relationship. This analysis quantified the concordance of disease prevalence between OFH and UKB and highlighted deviations from the overall trend. We further compared disease prevalence in OFH with that in the UK general population using estimates from the GBD for the UK (Fig. 2f and Supplementary Table 7). The same log–log framework was applied, correlating log_10_-transformed frequencies between OFH and GBD using Pearson’s *r* and fitting a log–log linear regression to describe the relationship. This comparison assessed how closely cohort-level disease frequencies in OFH align with population-level estimates.

To evaluate the reproducibility of known associations between health conditions and clinical correlates^29^, we estimated adjusted ORs for selected correlates in both OFH and UKB using logistic regression models of the form: outcome = correlate + age + sex + ethnicity + income (Supplementary information section 2.3). Self-reported female sex, white ethnicity and household income <£18,000 served as reference categories, with ‘false’ as the reference for self-reported diagnoses. Responses of ‘do not know’ and ‘prefer not to answer’ were retained as levels, whereas participants with missing ethnicity or income were excluded, leaving a maximum sample size of *N* = 1,897,796 (Supplementary Tables 3.1 and 3.2 for counts across analyses). Family history variables for autism, bipolar disorder and schizophrenia were defined by reports of the condition in a biological parent or sibling in OFH and by reports of that condition in a first-degree relative in UKB. UKB and OFH ORs were plotted with 95% confidence intervals (CIs) and Pearson’s *r* and log–log linear regression were computed between log_10_-transformed ORs.

Ethnicity-stratified estimates were restricted to ethnic groups in which case counts exceeded 1% of the analytic sample, providing a prespecified threshold for statistical stability; where confidence intervals are wide, stratified findings should be interpreted with caution and regarded as hypothesis-generating. The large number of participants from diverse ethnic groups is a strength of this study, enabling the detection of interaction effects that would probably be missed in smaller studies. However, several ethnic groups remain underrepresented, and for certain outcome–exposure combinations, stratified analyses could not be performed or were restricted to a subset of groups, a limitation that should be considered when interpreting and generalizing these findings.

We next modeled the association between current self-reported medication use and corresponding self-reported diagnoses in OFH using multivariable logistic regression adjusted for age, sex, ethnicity and income (reference categories of female, white ethnicity, household income <£18,000; ‘false’ as the reference for diagnosis and medication self-report) (Fig. 3c). Observations with missing self-reported ethnicity, income and medication responses or diagnosis responses were excluded, leaving a maximum *N* = 1,867,370 participants (see Supplementary Table 9.1 for counts across analyses). Analyses where it was possible to harmonize questionnaire versions 1 and 2 are noted in the Supplementary Information. To contextualize potential misclassification in self-reported phenotypes, we additionally compared selected self-reported diagnoses with linked inpatient EHR-derived diagnoses for several common chronic conditions, including cancer, asthma, type 2 diabetes, depression, hypertension, high cholesterol and osteoarthritis, using an unweighted Cohen’s kappa with a 95% confidence interval^62^ (Extended Data Fig. 6).

Finally, to characterize interdependencies among self-reported medication-use indicators, we computed pairwise correlations among medication-domain flags and derived aggregated medication usage-pattern variables (Fig. 3d). Binary–binary, binary–continuous and continuous–continuous relationships were quantified using the *φ* (phi), point–biserial and Pearson correlation coefficients, respectively, computed over the intersection of nonmissing participants (Supplementary Table 13). We used self-reported medication usage instead of dispensed medicines in primary care, as the latter were not yet available at the time of our analyses, whereas the former were available for the whole sample. Analyses were descriptive and exploratory in nature; effect estimates, correlations and regression coefficients are reported with corresponding 95% confidence intervals or *P* values where relevant. We did not model clustering within households or relatedness between participants in the present analyses. Regression models were used to summarize associations rather than to make causal inferences; accordingly, estimates should be interpreted as participant-level summaries. Researchers producing descriptive summary statistics with OFH need to use sampling weights that adjust for the population of the variables they examine, which OFH has announced it is actively developing, benchmarked to the 2021–2022 UK census^23^. Although we did not attempt any exploratory reweighting, the potential of weights to substantially alter the magnitude or direction of associations can be expected to vary by phenotype^63^ and research question. As with the UKB, although certain groups are underrepresented, they are probably still sufficiently large to enable reliable associations and disease risk^64^. However, this should be evaluated once appropriate weights become available.

Further analytic details, including model specifications, variable definitions and variable coding names are provided in the Supplementary Information and Supplementary Tables. Figures were generated in Visual Studio Code and subsequently refined for presentation using BioRender (https://www.biorender.com) and Inkscape (version 1.4.3).

### Reporting summary

Further information on research design is available in the Nature Portfolio Reporting Summary linked to this article.

## Online content

Any methods, additional references, Nature Portfolio reporting summaries, source data, extended data, supplementary information, acknowledgements, peer review information; details of author contributions and competing interests; and statements of data and code availability are available at https://doi.org/10.1038/s41591-026-04602-4.

## Supplementary information

Supplementary Information Supplementary Notes 1–3 and Tables 1–3.

Reporting summary

Supplementary Tables Supplementary Tables 1–16.

## Data Availability

This study makes use of both restricted and publicly available data. Access to data from OFH participants used in our analysis is restricted to registered researchers who make a successful study application to the OFH Access Board. Any researcher from around the world who works for, or are a supervised student of, universities, charities, health services or organizations involved in health research can apply to access the data by applying to become a ‘registered researcher’ and submitting a study application at https://research.ourfuturehealth.org.uk/apply-to-access-the-data/. Access to data from the UKB is also restricted; interested researchers should register to apply for access on the UKB website (https://www.ukbiobank.ac.uk/use-our-data/apply-for-access/). The data held by OFH used in this study were approved under the OFH study ID 240228; the data held by the UKB were approved under the UKB project ID 597553. Publicly available data came from the following sources: OFH map of clinic locations (https://go.nature.com/41oKFic); HSE 2021 and 2022 (https://digital.nhs.uk/data-and-information/publications/statistical/health-survey-for-england); ONS 2021 census data for England and Wales (https://www.nomisweb.co.uk/sources/census_2021); ONS mid-2023 population estimates for the UK (https://www.ons.gov.uk/peoplepopulationandcommunity/populationandmigration/populationestimates/bulletins/annualmidyearpopulationestimates/mid2023); National Records of Scotland 2022 census data (https://www.scotlandscensus.gov.uk/); prevalence of 15 major diseases in Finngen^6^; All of Us data snapshots (https://www.researchallofus.org/data-tools/data-snapshots/); GBD 2021 (https://vizhub.healthdata.org/gbd-results/); and NDRS cancer prevalence for 2022 (https://digital.nhs.uk/ndrs/data/data-outputs/cancer-data-hub). We provide the full deidentified, processed summary-level data used to create all figures in the Supplementary Tables and full details of the publicly available data sources used in each figure in the Supplementary Information. All publicly available datasets^59^ we use are available via Zenodo at https://doi.org/10.5281/zenodo.21038180 (ref. ^65^).

## Extended data

**Extended Data Table 1:** Our Future Health target cohort composition by age, sex, and ethnicity

	Female				Male			
	White	Black	Asian	Mixed/Other	White	Black	Asian	Mixed/Other
Age								
18–19	53,777	3,829	7,887	4,857	55,937	3,982	8,321	5,119
20–29	315,271	17,695	42,515	23,896	311,381	16,680	41,401	22,979
30–39	346,102	18,819	52,808	23,453	331,548	15,258	45,968	21,563
40–49	324,853	18,766	44,357	17,012	315,509	15,479	43,073	16,625
50–59	385,993	17,240	26,585	11,888	372,458	15,370	25,952	11,841
60–69	319,702	7,820	17,574	6,267	305,820	7,004	16,356	6,170
70–79	273,138	3,270	9,276	3,002	245,331	2,128	7,292	2,712
80–84	85,176	1,396	2,559	750	66,277	997	2,351	635
85+	92,097	1,182	1,984	739	54,079	794	1,611	462
*N*	2,196,109	90,017	205,544	91,865	2,058,341	77,693	192,325	88,107
%	43.9%	1.8%	4.1%	1.8%	41.2%	1.6%	3.8%	1.8%

Adapted from Table 1 in the Our Future Health Protocol, available alongside additional information on the sample ambition at: https://ourfuturehealth.org.uk/our-research-mission/how-our-future-health-works/.

**Extended Data Table 2:** Our Future Health recruitment numbers by age, sex, ethnicity, and deprivation for different data releases

Total	Consented participants^*^ (3 June 2025)		Completed questionnaire^*^ (11 Jun 2025 data release 11)		Completed questionnaire (17 Sep 2025 data release 12)		Completed questionnaire (11 Dec 2025 data release 13)		UK Census 2021/2022
	N	%	N	%	N	%	N	%	%
	2,461,601	100.0	1,859,888	100.0	1,900,495	100,0	1,929,744	100.0	-
Age group (years)									
18–29	246,385	10.0	164,404	8.8	180,631	9.5	183,950	9.5	18.7
30–39	411,695	16.7	272,543	14.7	282,724	14.9	287,511	14.9	17.1
40–49	430,370	17.5	293,262	15.8	300,678	15.8	305,937	15.9	15.9
50–59	507,595	20.6	390,512	21.0	406,938	21.4	413,188	21.4	17.3
60–69	512,390	20.8	432,567	23.3	436,306	23.0	442,302	22.9	13.7
70–79	295,451	12.0	258,746	13.9	250,951	13.2	253,972	13.2	11
≥80	57,715	2.3	47,854	2.6	42,267	2.2	42,884	2.2	6.3
Sex at birth									
Female	1,147,547	46.6	1,065,345	57.3	1,088,628	57.3	1,105,287	57.3	51.7
Male	851,324	34.6	793,428	42.7	810,707	42.7	823,244	42.7	48.3
Other	1,487	0.1	1,115	<0.1	1,160	<0.1	1,213	<0.1	-
Unknown	461,243	18.7	-	-	-	-	-	-	-
Ethnicity									
Asian	113,315	4.6	100,195	5.4	103,228	5.4	106,509	5.5	7.9
Black	33,130	1.3	28,189	1.5	29,212	1.5	29,940	1.6	3.3
White	1,772,179	72	1,673,311	90.0	1,708,109	89.9	1,731,955	89.7	85.1
Mixed	35,619	1.4	32,224	1.7	33,123	1.8	33,821	1.8	1.8
Other	30,600	1.2	25,969	1.4	22,830	1.2	23,400	1.2	1.9
Unknown	476,758	19.4	-	-	3,992	0.2	4,118	0.2	-
IMD ^†^									
1 - most deprived	321,738	13.1	221,139	11.9	178,086	13.0	177,056	12.9	20
2	402,687	16.4	292,611	15.7	234,375	17.1	233,684	17.0	20
3	458,793	18.6	347,752	18.7	273,355	20.0	272,798	19.9	20
4	551,194	22.4	428,467	23.0	314,540	22.9	314,926	23.0	20
5 - least deprived	652,738	26.5	518,544	27.9	366,498	26.7	366,659	26.7	20
Unknown	74,451	3.0	51,375	2.8	6,841	0.5	6,699	0.5	-

Selected characteristics of participants for different recruitment stages, from consented to full participant (completed questionnaire and usable blood sample). Age group calculated for recruitment numbers based on year of birth and for data release based on month and year of birth. Combined census data for England, Scotland, Wales, and Northern Ireland from 2021 and 2022 for adults (>18 years). A dash indicates that data are not available or do not exist in this category. IMD, Index of Multiple Deprivation. ^***^Figures are adapted from^20^. ^†^Absolute numbers for data release 12 and 13 do not sum to total and are lower than for data release 11 due to differences in data source used; data for releases 12 and 13 come from linked health records as in Fig. 1, data for release 11 is adapted from^20^, which relies on restricted IMD data that is currently not part of the baseline phenotypic data release.

## Code availability

We built the custom Python (https://www.python.org/) library Phenofhy introduced in ref. ^51^ to process and analyze OFH phenotypic data, which is currently available via GitHub at https://github.com/studiovincentstraub/phenofhy, and will be made available on PyPI. Phenofhy is written in Python 3.9.21 and relies on several additional libraries. It is open-source, and a beta version is readily available for other registered researchers to upload and use on the OFH TRE^51^, DNAnexus. The code written to undertake all analyses on DNAnexus and create plots for this study, which made use of Phenofhy, Python 3.9.21 and R v4.0.3 (https://www.r-project.org/), is available in form of Jupyter Notebooks via GitHub at https://github.com/studiovincentstraub/article-our-future-health-phenotypic-data.
