# Supplementary material for: Phenomic profiles and disease patterns of 1.9 million participants from Our Future Health
Source: Nat Med. 2026 Sep 10;32(10):3899–910. doi: 10.1038/s41591-026-04602-4 (PMC13668653; doi:10.1038/s41591-026-04602-4)
Supplement: Supplementary file 1 [file 41591_2026_4602_MOESM1_ESM.pdf]

nature medicine

Supplementary information https://doi.org/10.1038/s41591-026-04602-4

Phenomic profiles and disease patterns of 1.9 million participants from Our Future Health

In the format provided by the authors and unedited

Scope of this Supplementary Information

This Supplementary Information provides methodological detail that complements the main **Methods** section. It includes an additional box of key biases, extended footnotes for main-text figures and tables, descriptions of supplementary tables, additional results from secondary analyses, additional tables, and documentation of phenotype and variable mappings used for cross-cohort comparisons.

Supplementary Table of Contents

Supplementary Note 1. Box 1 and extended footnotes for main text and display items

1.1 Box 1
1.2 Table 1 (Main Text)
1.3 Figure 2 (Main Text)
1.4 Figure 3 (Main Text; Extended Data Fig. 3)

Supplementary Note 2. Supplementary table descriptions

2.1 Supplementary Tables 1
2.2 Supplementary Tables 2
2.3 Supplementary Tables 3
2.4 Supplementary Tables 4
2.5 Supplementary Tables 5
2.6 Supplementary Tables 6
2.7 Supplementary Tables 7
2.8 Supplementary Tables 8
2.9 Supplementary Tables 9
2.10 Supplementary Tables 10
2.11 Supplementary Tables 11
2.12 Supplementary Table 12
2.13 Supplementary Table 13
2.14 Supplementary Table 14
2.15 Supplementary Table 15
2.16 Supplementary Table 16

Supplementary Note 3. Phenotype and variable mappings

3.1 Our Future Health questionnaire versions
3.2 Our Future Health–UK Biobank mapping
3.3 Our Future Health–Global Burden of Disease mapping
3.4 Our Future Health–Health Survey for England mapping

Supplementary Information Tables 1-3

Supplementary Note 1: Extended footnotes for main text and display items

Supplementary Note 1.1: Box 1

Known biases and data limitations of volunteer-based biobanks and cohort studies

Large-scale volunteer biobanks are subject to systematic biases and data limitations related to recruitment design, sampling, and surveying. These can affect their representativeness, distort prevalence estimates, and impact exposure–disease outcome associations[9]. Below we highlight a number of biases found in existing population-scale biobanks that may need to be further investigated in the OFH cohort once recruitment is complete.

- **Selection bias** occurs when enrolled participants differ systematically from non-participants in characteristics related to the exposures or outcomes under study, such that inferences from the sample may not generalise to the target population.
- **Healthy volunteer bias** is a well-documented consequence of self-selection, in which volunteers are systematically healthier than the general population. In the case of the UK Biobank (UKB), participants are less likely to be obese, smoke, or drink alcohol daily, and all-cause mortality among those aged 70–74 has been shown to be approximately half that of the general population[15]. Relatedly, higher screening intensity, whereby health-engaged participants undergo more diagnostic investigation, can also inflate detection of certain conditions and reduce prevalence of conditions concentrated in health-avoidant groups.
- **Sex-differential participation bias** refers to systematic over- or underrepresentation based on participant sex. For instance, the Estonian Biobank, despite covering approximately 20% of the population, shows overrepresentation of younger, female participants relative to regional expectations[7].
- **Survivorship bias** arises because participation requires a level of health or functioning that excludes individuals with the most severe or fatal conditions, leading to underrepresentation of advanced disease. It is related to mortality selection, which occurs when a non-random subset of a population of interest has died before data collection and is unobserved in the data.
- **Collider bias** occurs when exposure and outcome influence a third variable that is impacted by study design creating a spurious association[43,44]. In cases where participation has genetic components, collider biases can cause skewed associations and unreliable effect estimates in genome-wide association studies.
- **Genetic participation bias** arises because participation itself has been shown to have a heritable component[47], meaning that allele frequencies and genotype–phenotype associations estimated in biobanks may not generalise to the broader population. As a result, estimates of heritability and genetic correlation derived from volunteer biobanks require careful interpretation (see **Box 1** in[21]).
- **Recall bias** refers to systematic error in self-reported data arising from imperfect memory or social desirability effects. Non-random-missingness and item-level non-response behaviours (e.g., "prefer not to answer", "I don't know") have been found to be systematic, partially heritable, and cluster around stigmatised questions (e.g., “does your partner complain about your snoring?”), and can similarly induce collider bias in downstream analyses[9].

Sample ascertainment differences, whereby different data sources (e.g., self-report and electronic health records) capture different fractions of true disease burden with different sensitivity and specificity can further produce apparent prevalence differences independently of participation bias. Importantly, distortions in prevalence estimates and non-representativeness does not always reflect selection bias and need not be a problem, as it can be the result of deliberate recruitment design choices or other sampling mechanisms. Enriching for disease cases through hospital-based recruitment, as in the case of the Million Veteran Program[26], for example, can be an intentional strategy to maximise power to detect genetic effects in case-control studies.

Supplementary Note 1.2: Table 1

Sex assigned at birth in Our Future Health (OFH) was self-reported at baseline by participants using the options female (N = 1,058,703), male (N = 787,521), other/intersex (N

= 139), and “prefer not to answer” (*N* = 989). Participants reporting other/intersex or “prefer not to answer” were excluded from sex-stratified analyses shown in **Table** 1, which compared male and female participants in OFH to Health Survey England (HSE).

Clinic measurements for height, weight, and waist circumference allowed broad capture ranges at data entry (height: 90–299 cm; weight: 20–400 kg; waist circumference: 30–200 cm). To reduce the influence of implausible values, additional thresholds were applied: height >240 cm excluded, weight >250 kg excluded, and waist circumference >180 cm excluded. These exclusions affected fewer than 0.02% of participants with clinic measurements.

For *current health status* and *any long-standing physical or mental health condition or illness*, the category “unknown” combines responses of “do not know” and “prefer not to answer.”

Comparisons with HSE used 5,774 HSE respondents aged ≥18 years (*N* = 3,127 females; *N* = 2,647 males). For categorical variables, “unknown” combines “don’t know” and “refuse to answer.” For continuous variables, denominators exclude respondents for whom the question was not applicable (e.g., smoking initiation among never smokers). Waist circumference was measured only among HSE respondents attending a nurse visit.

Supplementary Note 1.3: Figure 2

Self-reported housing-related variables had 62 missing responses; 21,208 participants selected “prefer not to answer” for housing relatedness. Country-of-birth responses included 3,321 “UK (don’t know country),” 6,548 “prefer not to answer,” and 647 “do not know.” These categories are displayed in **Fig. 2** and **Supplementary Tables 4.1–4.2** for completeness.

Supplementary Note 1.4: Figure 3

Conditions with fewer than 50 cases or prevalence <0.001% were excluded from prevalence comparisons (**Fig. 3a–c; Extended Data Fig. 3**) to avoid unstable estimates driven by sparse counts or poorly matched phenotypes.

As stated in the **Results**, Comparisons between OFH and Global Burden of Disease (GBD) estimates (**Fig. 3b; Supplementary Table 7**) are exploratory in nature. OFH collects lifetime self-reported diagnoses, whereas GBD reports point prevalence. Absolute prevalence values are therefore not directly comparable, particularly for acute or episodic conditions. Only chronic or long-duration conditions were retained. Observed differences may additionally reflect population structure, diagnostic practices, access to care, and reporting behaviour and results should therefore be interpreted with according caution.

Supplementary Note 2: Supplementary table descriptions

2.1: Supplementary Table 1

Case counts and prevalence estimates for FinnGen (FG), UKB, which is included in **Supplementary Table 1** as an additional population-scale comparison, and All of Us (AoU) were obtained from existing published and online resources, including Table S7 in Kurki et al. (2023); the FinnGen R5 endpoint (https://r5.risteys.finngen.fi/) and results browsers (https://results.finngen.fi/); the AoU Data Browser (https://databrowser.researchallofus.org/ehr/conditions/); and the UKB Research Analysis Platform. Access to UKB and OFH data was obtained via approved study applications, as described in the main **Methods** section. As described in Kurki et al. (2023), SNOMED to International Classification of Diseases 10th revision (ICD-10) mappings for comparator cohorts were generated using the IHTSDO mapping tool. OFH case definitions were derived from admitted-patient-care records using ICD-10 codes, as detailed in **Supplementary Table 1**. Dataset-specific filters including cancer-registry linkage, sex restrictions, and main-diagnosis flags were applied where appropriate.

2.2: Supplementary Table 2

For each condition, counts reflect participants who both self-reported the corresponding diagnosis and self-reported regular use of one or more relevant medications or treatments. Participants selecting “prefer not to answer,” “do not know,” “other not listed,” or “none of the above” were excluded from self-reported medication-use analyses.

Alzheimer’s disease includes participants reporting Alzheimer’s disease or dementia, excluding early-onset forms.

Breast cancer medication use includes participants self-reporting malignant neoplasm of the breast who selected one or more of the following treatments: chemotherapy, hormone therapy, or immunotherapy/targeted therapy.

Type 2 diabetes medication use includes participants self-reporting type 2 diabetes who selected one or more of the following medications: acarbose; DPP-4 inhibitors (e.g. sitagliptin, vildagliptin, saxagliptin, alogliptin); GLP-1 receptor agonists (e.g. exenatide, liraglutide, lixisenatide); insulin; metformin; prandial glucose regulators (e.g. repaglinide, nateglinide); SGLT2 inhibitors (e.g. dapagliflozin, canagliflozin, empagliflozin, ertugliflozin); sulphonylureas (e.g. glibenclamide, gliclazide, glipizide, tolbutamide); and thiazolidinediones (e.g. pioglitazone).

Alzheimer’s disease medications include acetylcholinesterase inhibitors (e.g. donepezil, galantamine, rivastigmine) and memantine.

Medication-based endpoints were derived from self-reported regular use of disease-specific drugs, providing a complementary indicator of treatment exposure where linked prescription data were unavailable. Because some medications may be prescribed for other indications (e.g. metformin for polycystic ovary syndrome, GLP-1 receptor agonists for obesity), some endpoint misclassification is possible.

Condition-specific notes:

- Glaucoma: OFH values reflect glaucoma in general; comparator sources use primary open-angle glaucoma.
- Rheumatoid arthritis: OFH values reflect rheumatoid arthritis in general.
- Atrial fibrillation/flutter: In OFH, atrial fibrillation and flutter are classified as *Abnormal Heart Rhythm (Arrhythmia)*; comparator sources use ICD-10 I48.9.
- Type 2 diabetes medications: Estimates exclude insulin and statins; totals are tallied across all other medication categories.
- Type 1 diabetes medications: Restricted to participants taking insulin.
- Depression medications: Estimates exclude certain antidepressants (e.g. mirtazapine, venlafaxine, duloxetine).
- Breast/prostate cancer medications: Restricted to participants with a diagnosis of breast or prostate cancer, respectively.

2.3: Supplementary Tables 3.1 and 3.2

For both diagnosis and medication self-report, *false* was treated as the reference category. Self-reported Female sex assigned at birth, White ethnicity, and household income <£18,000 served as reference categories. Responses of "do not know" and "prefer not to answer" were retained as analytic categories. Participants with missing ethnicity (n = 898) or income (n = 6,008) were excluded. The number of observations used in each model is reported in the tables. Only questionnaire version 2 data were used.

Family-history variables were defined using participant self-reports indicating whether a biological mother, father, or sibling had received a clinical diagnosis of autism, bipolar disorder, or schizophrenia.

Logistic regression models followed the specification:

$$outcome \sim correlate + age + sex + ethnicity + income$$

Stratification by self-reported ethnicity was performed in **Table 3.2**. For each health outcome, associations with selected clinical correlates were estimated using logistic regression models of the specification:

$$outcome \sim correlate \times ethnicity + age + sex + income$$

Effect heterogeneity by self-reported ethnicity was assessed by comparing this model with an extended model additionally including an interaction term between the clinical correlate and ethnicity. Likelihood-ratio tests were used to evaluate the statistical significance of the interaction. Where P = 0, P values were <1E-300. Ethnicity-stratified odds ratios were estimated only for associations with a statistically significant interaction test. To ensure adequate statistical stability, stratified estimates were further restricted to ethnic groups with case counts exceeding 1% of the analytic sample for the corresponding outcome. Associations not meeting these criteria are not reported.

Supplementary **Table 3.2** summarises likelihood-ratio tests for interaction between selected established clinical correlates and self-reported ethnicity across health conditions in OFH, with UKB results shown for comparison. Evidence of heterogeneity by ethnicity was observed for several associations. Strong interaction effects were detected for anxiety in relation to alcohol consumption (χ^2=2391, df=5, p<0.001) and obsessive–compulsive disorder (χ^2=88.2, df=5, p<0.001), as well as for attention-deficit/hyperactivity disorder in relation to anxiety (χ^2=173.7, df=5, p<0.001), indicating that the strength of these associations differed across ethnic groups. Significant interaction was also observed for obesity in relation to back pain (χ^2=38.2, df=5, p<0.001), gastro-oesophageal reflux disease (χ^2=32.3, df=5, p<0.001), and osteoarthritis (χ^2=14.4, df=5, p=0.013), and for smoking in relation to cataracts (χ^2=33.2, df=15, p=0.004).

No evidence of interaction by ethnic group was observed for several condition–exposure pairs. Specifically, interactions were non-significant for ischaemic heart disease and hypertension in the OFH 40–69 subgroup (P = 0.23), and for hypertension and obesity in UKN (P = 0.15). For dementia, no significant interactions were detected with heart failure (UKB P = 0.88; OFH P = 0.35; OFH 40–69 P = 0.81) or with depression (UKB P = 0.98; OFH P = 0.22; OFH 40–69 P = 0.18).

Similarly, no interaction was observed for back pain and obesity in UKB (P = 0.39). For lung cancer, interactions with smoking status were non-significant across datasets (UKB P = 0.07; OFH P = 0.43; OFH 40–69 P = 0.51), as were interactions with family history of lung cancer (UKB P = 0.75; OFH P = 0.12; OFH 40–69 P = 0.12).

For colorectal (bowel) cancer, no significant interactions were detected with alcohol consumption (UKB P = 0.07; OFH P = 0.34; OFH 40–69 P = 0.50) or with obesity (UKB P = 0.69; OFH P = 0.63; OFH 40–69 P = 0.86). Interactions between ischaemic heart disease and anaemia were also non-significant in OFH (P = 0.48) and in the OFH 40–69 subgroup (P = 0.67). Finally, no significant interaction was observed between schizophrenia and family history of schizophrenia (UKB P = 0.17; OFH 40–69 P = 0.06).

Abbreviations used in **Supplementary Tables 3.1-3.2:** OFH, Our Future Health; UKB, UK Biobank; ADHD, attention-deficit/hyperactivity disorder; OCD, obsessive-compulsive disorder.

2.4: Supplementary Tables 4.1-4.3

Supplementary Tables 4.1-4.2

Percentages are calculated among all participants asked the question. Responses of “prefer not to answer” and missing data were excluded from the table but retained in the denominator; as such, totals may not sum to 100%.

In OFH, for the variable, *Any long-standing physical or mental health condition or illness*, the question was asked only in questionnaire version 2, and percentages are reported with respect to the full cohort. BMI measurements rely on clinic measurements data; consequently, BMI distributions do not sum to 100% because only 1,433,275 out of 1,900,557 participants (75.4%) in data release 12 had clinic measurements.

Importantly, the multiple and differential impacts of the Coronavirus Disease 2019 (COVID-19) pandemic on both participant self-reporting of health-related phenotypes and actual health-related behaviours need to be considered (see **Discussion**). This applies both to interpreting OFH data and HSE data (see **Supplementary Information Table 1** for comparison of data collection methods), especially as data collection in the 2021 HSE differed from previous years to limit contact between participants and interviewers (HSE 2021) and national surveys suggested as much as 80% of adults planned to modify their lifestyle in 2021 due to COVID-19 (https://digital.nhs.uk/data-and-information/publications/statistical/health-survey-for-england).

OFH variable names

Female and male are based on self-reported sex at birth (DEMOG_SEX_1_1 in questionnaire version 1; DEMOG_SEX_2_1 in questionnaire version 2). Participants reporting “Other” (version 1) or “Intersex” (version 2) were excluded (<0.01%).

Alcohol consumption (ALCOHOL_CURR_1_1); e-cigarette use (SMOKE_VAPE_1_1 in version 1; SMOKE_TOBACCO_TYPE_1_M in version 2); current health status (HEALTH_STATUS_CURR_1_1); long-standing physical or mental health condition (HEALTH_STATUS_CHRONIC_1_1); height (HEIGHT); weight (WEIGHT); waist circumference (WAIST).

Obesity was defined as BMI ≥30 kg/m². Physical activity was derived from ACTIVITY_WALK_DAYS_2_1. Smoking status was derived using questionnaire-specific logic for versions 1 and 2, as described in **Methods**.

HSE variable names

Smoking status (cigsta3_19); e-cigarette use (eciguse2_19); current health status (genhelf4, with HSE “very good” mapped to OFH “excellent”); long-standing condition (ill12m); physical activity (derived from daywlk); BMI (bmisr_adj); height (height_adj); weight (weight_adj); waist circumference (wstval).

In HSE, BMI, height and weight values were adjusted based on self-reported responses in the individual survey. Waist circumference was measured and is only available for participants who attended the nurse visit.

Abbreviations used in **Supplementary Tables 4.1-4.2:** OFH, Our Future Health*; HSE,* Health Survey for England*;* N, number of participants (excluding missing data); %, percentage of participants in the category; SD, standard deviation; BMI, body mass index; WC, waist circumference. HSE data were weighted using survey weights; further details are provided in the HSE 2021 User Guide (https://digital.nhs.uk/data-and-information/publications/statistical/health-survey-for-england/2021)

Supplementary Table 4.3

Heart measurements taken at clinic appointments of participants in data release 13 (released on 11 December 2025). *Based on reported sex assigned at birth. Participants

who reported “Other”, “Intersex” or “Prefer not to answer” are not shown. [†]Third readings for heart measures are obtained only in v2 of the clinic measurements process and if at least one of the following criteria is met: SBP readings 1 and 2 are either both greater than or equal to 160 mmHg or both less than 90 mmHg; or Heart rate readings 1 and 2 are either both greater than or equal to 100 bpm or both less than 60 bpm, or Heart rhythm readings 1 and 2 are both “irregular”.

2.5: Supplementary Table 5

Data were obtained from UKB and OFH via the approved study applications described in **Methods**. HSE data were obtained from Table S2 in the Adult Health table of HSE 2022 (https://digital.nhs.uk/data-and-information/publications/statistical/health-survey-for-england/2022-part-2/health-survey-for-england-hse-2022-part-2-data-tables).

Percentages were calculated among participants providing meaningful responses. Non-informative responses (e.g., “prefer not to respond,” “don’t know”) were excluded from both numerator and denominator. For *any long-standing physical or mental health condition or illness*, the question was asked only in questionnaire version 2. Counts and frequencies for all traits except cancer were computed among participants with non-missing values who completed questionnaire version 2 (N = 1,847,466).

Abbreviations used in **Supplementary Tables 5**: OFH, *Our Future Health*; N, number of participants (excluding missing data); %, percentage of participants; OFH coding name, questionnaire section identifier corresponding to self-reported health condition; UKB data field, UKB section identifier corresponding to self-reported health condition.

2.6: Supplementary Table 6

Data were obtained from UKB and OFH via the approved study applications described in the main **Methods** section. Analyses were restricted to self-reported diagnoses of disease and health conditions to maximise comparability. Participants reporting “none,” “don’t know,” or “prefer not to respond” were excluded.

Code mapping between OFH and UKB questionnaire fields was performed using a structured, multi-stage curation process. Initial candidate matches were generated by comparing field descriptions across datasets. All candidate matches were then manually reviewed, and only clinically equivalent mappings were retained. OFH fields without suitable UKB counterparts were excluded.

Abbreviations used in **Supplementary Tables 6:** OFH, Our Future Health; N, number of participants (excluding missing data); %, percentage of participants; OFH coding name, questionnaire section identifier; UKB data field, UK Biobank section identifier.

2.7: Supplementary Table 7

Data were obtained from OFH via the approved study applications described in **Methods** and the GBD (Global Burden of Disease Collaborative Network 2022) restricted to estimates for the United Kingdom for 2021.

OFH diagnosis codes and GBD causes were manually mapped on a one-to-one basis by systematically comparing field names, definitions, and underlying clinical concepts. Mappings were evaluated for clinical equivalence and conceptual alignment. Mappings were rejected where aggregation or mismatch in disease scope would be expected to distort prevalence estimates (for example, mapping GBD “chronic myeloid leukaemia” to the broader OFH category “leukaemia”). Mappings were accepted where definitions differed in wording or coding granularity but referred to the same underlying clinical condition (for example, mapping OFH “ectopic or tubal pregnancy” to GBD “ectopic pregnancy”). Only mappings meeting these criteria were retained for cross-dataset prevalence comparisons.

Abbreviations used in **Supplementary Tables 7:** OFH, Our Future Health; N, number of participants (excluding missing data); %, percentage of participants; OFH coding name; GBD cause.

As discussed in Straub et al. (2025), alongside the need for additional prevalence comparisons using linked health records, accurately harmonizing OFH questionnaire data with external datasets will require the development of standardized mappings.

2.8: Supplementary Tables 8

Analyses include only participants who completed questionnaire version 2. Individuals aged under 18 were excluded.

2.9: Supplementary Tables 9.1 and 9.2

Total N = 1,867,370. Missing self-reported ethnicity, missing income, missing medication data, and missing diagnosis data were excluded.

Logistic regression models followed the specification:

current_medication_use ~ ever_diagnosis + age + sex + ethnicity + income

Reference categories were self-reported female sex, White ethnicity, household income <£18,000, and *false* for diagnosis and medication self-report. Responses of “do not know” and “prefer not to answer” were retained as analytic categories.

Stratification by ethnicity was performed in **Table 9.2**. For each medication, associations with selected diagnoses were estimated using logistic regression models of the specification:

current_medication_use ~ ever_diagnosis x ethnicity + age + sex + income

Effect heterogeneity by ethnicity was assessed by comparing this model with an extended model additionally including an interaction term between the *ever_diagnosis* variable and (self-reported) ethnicity. Likelihood-ratio tests were used to evaluate the statistical significance of the interaction. Where P = 0, P values were <1E-300. Ethnicity-stratified odds ratios were estimated only for associations with a statistically significant interaction test. To ensure adequate statistical stability, stratified estimates were further restricted to ethnic

groups with case counts exceeding 1% of the analytic sample for the corresponding outcome. Associations not meeting these criteria are not reported.

Evidence of interaction by ethnicity was observed for multiple medication–diagnosis pairs. Significant heterogeneity was detected for autoimmune medication use in relation to autoimmune disorders (χ^2=16.8, df=5, p=0.0049) and for cancer-related medication use in relation to cancer diagnoses (χ^2=20.0, df=5, p=0.0012). Strong evidence of interaction was also observed for medications relating to heart or circulatory conditions (χ^2=60.5, df=5, p<0.001), endocrine disorders (χ^2=38.6, df=5, p<0.001), digestive system or liver problems (χ^2=137.7, df=5, p<0.001), and neurological disorders (χ^2=139.3, df=5, p<0.001), indicating that the strength of the association between medication use and diagnosis differed across ethnic groups. Ethnicity-specific odds ratios are presented only for associations with statistically significant interaction tests and where case counts were appropriate, consistent with the prespecified analytic approach. Cancer and neurological associations were not reported as case counts were less than 1%.

2.10: Supplementary Tables 10.1 and 10.2

Population estimates were obtained from the Office for National Statistics (ONS) mid-2023 population estimates for Great Britain (England, Scotland and Wales) (https://www.ons.gov.uk/peoplepopulationandcommunity/populationandmigration/populationestimates/datasets/populationestimatesforukenglandandwalesscotlandandnorthernireland). Ethnicity data were obtained from the 2021 Census for England and Wales (https://www.ons.gov.uk/peoplepopulationandcommunity/culturalidentity/ethnicity/datasets/ethnicgroupbyageandsexinenglandandwales) and the 2022 Scotland Census (https://www.scotlandscensus.gov.uk/).

2.11: Supplementary Table 11

Cancer prevalence estimates for England were obtained from the NHS England National Disease Registration Service (NDRS), Cancer Prevalence in England, 2022 (https://digital.nhs.uk/ndrs/data/data-outputs/cancer-data-hub/cancer-prevalence?utm_source=chatgpt.com).

Data represent observed, limited-duration prevalence for individuals diagnosed between 1995 and 2022 who were alive on 31 December 2022. Counts and crude rates per 100,000 population are based on 2022 mid-year ONS population estimates. “Lung cancer” refers to tumours of the trachea, bronchus, and lung (ICD-10 C33–C34), aligning with the “lung or bronchial” category used in OFH analyses. Analyses were restricted to OFH participants aged 20 years or older who registered in England prior to June 2024.

2.12: Supplementary Table 12

This table contains the pairwise Pearson correlation matrix between all derived medication domain flags and higher-level medication usage–pattern variables generated from the OFH cohort. Correlations are shown for the full cohort and stratified by age group (18–29, 30–59, and ≥60 years). The correlation matrix characterizes co-use and dependency patterns among medication classes and aggregated usage indicators across participants. It was

designed to capture shared medication behaviours, polypharmacy trends, and system-level diversity of treatment exposure within the cohort.

Two categories of variables are included (see **Supplementary Information Table 2** for details):

1. Medication domain flags: All variables beginning with derived.medicates_, representing binary (0/1) indicators of regular use within specific pharmacological or physiological domains.
2. Aggregated usage-pattern variables: Higher-level system or summary measures derived from grouped medication domains, capturing overall medication burden and diversity of use.

All pairwise correlations were computed using Pearson's correlation coefficient (*r*) on the intersection of non-missing participants for each variable pair. For binary–binary variable pairs, Pearson's *r* is equivalent to the Phi coefficient. For binary–continuous pairs, Pearson's

r is equivalent to the point–biserial correlation. For continuous–continuous pairs, Pearson's *r* corresponds to the standard Pearson correlation coefficient. Variables with zero variance were assigned a correlation value of 0.

2.13: Supplementary Table 13

This table compares several features of Our Future Health to other large-scale cohort programmes, including AoU, China Kadoorie Biobank (CKB), Million Veterans Program, and the UKB. Data were obtained from the programme URLs, available in **Supplementary Table 13** and Gallagher et al. (2025). Information on the funding of each cohort was confirmed using the following additional sources: All of Us (https://allofus.nih.gov/article/program-overview); U.S. Department of Affairs Million Veterans Progam (https://www.mvp.va.gov/pwa/); FinnGen (https://www.finngen.fi/en/funding); China Kadoorie Biobank (https://www.ckbiobank.org/about-us/funding); UK Biobank (https://www.ukbiobank.ac.uk/about-us/our-funding/).

Further information is available on each of the respective programme URLs, and additional information on phenotypic measurements and available biospecimens for AoU, CKB, UKB and other cohorts is provided by the Richard Doll CONSORTIUM available at: https://www.richarddollconsortium.org/search-the-studies.

2.14: Supplementary Table 14

Prevalence of self-reported health behaviours, mental health diagnoses, and medication use stratified by recruitment period in Our Future Health (OFH). Participants recruited in 2022–2023 (N = 889,207) and 2024 onwards (N = 1,010,863) are shown. For health behaviours and medications, the denominator is all participants with a non-missing response. For mental health diagnoses, the denominator reflects participants who responded to the relevant diagnostic question and selected at least one option (i.e. excluding "none of the above", "prefer not to answer", and "do not know"). Antidepressant, antipsychotic, and contraceptive denominators reflect participants who were asked the relevant specific medication sub-

question. Because OFH recruitment began in 2022, entirely after the COVID-19 pandemic onset, no pre-pandemic baseline is available for direct comparison. This table is presented to examine whether prevalence estimates vary systematically across recruitment waves as a proxy indicator of potential pandemic-related temporal bias in outcome reporting. Prevalence estimates are broadly stable across periods, providing reassurance that findings are not materially driven by differential reporting related to the timing of recruitment.

2.15: Supplementary Table 15

To evaluate the reliability of self-reported (SR) diagnoses used in cross-cohort comparisons, we compared SR phenotypes with linked inpatient electronic health record (EHR)-derived diagnoses for several common chronic conditions in OFH, including cancer, asthma, type 2 diabetes, depression, hypertension, high cholesterol, and osteoarthritis, shown in **Supplementary Table 15**. Analyses were restricted to participants with both completed questionnaire data and linked inpatient EHR records available (N = 1,371,821). For each condition, we quantified the number of participants identified from SR only, EHR only, both sources, or either source, and calculated unweighted Cohen's κ statistics with bootstrap-derived 95% confidence intervals to assess agreement beyond chance. Agreement varied substantially across conditions, ranging from κ = 0.29 for high cholesterol to κ = 0.66 for cancer. Moderate agreement was observed for asthma (κ = 0.52), hypertension (κ = 0.51), and type 2 diabetes (κ = 0.57), whereas lower agreement was observed for osteoarthritis (κ = 0.34) and depression (κ = 0.37).

Systematic differences between SR- and EHR-derived prevalence estimates were also evident. EHR-derived prevalence exceeded SR prevalence for several conditions commonly recorded during inpatient care or as comorbidities, including hypertension and osteoarthritis. Conversely, SR prevalence exceeded inpatient EHR prevalence for depression, likely reflecting under-ascertainment of ambulatory mental health conditions in hospital-based records. High cholesterol showed relatively balanced discordance but comparatively low agreement overall, consistent with differences in self-awareness, clinical coding practices, and healthcare setting. Overall, these findings indicate moderate concordance between SR and inpatient EHR-derived phenotypes, with variation across conditions reflecting expected differences in disease salience, coding practices, and ascertainment mechanisms rather than systematic error in a single data source. ICD-10 code definitions used to derive EHR phenotypes are provided in **Supplementary Information Table 3.**

2.16: Supplementary Table 16

This table provides the ICD-10-to-ORPHA code consensus mapping used to define rare and low-prevalence conditions, adapted from Patrick et al. (2022). For each condition, we report the disease name, ORPHA code, corresponding ICD-10 codes, and disease group classification based on the ICD-10 chapter structure. Case counts are provided for both OFH and UKB cohorts, together with sex-stratified proportions for UKB participants. Prevalence estimates can be derived from these counts using the cohort denominators reported in the main text. The analysed set comprised N = 187 conditions selected from the N = 420 conditions previously characterised in UKB by Patrick et al. (2022). Conditions were restricted to those that could be robustly ascertained in both cohorts and that met OFH statistical disclosure control requirements (≥10 cases). The analysed set includes clinically

defined rare and ultra-rare diseases, together with selected low-prevalence conditions retained for comparative purposes.

Consistent with the conservative consensus framework of Patrick et al. (2022), only ICD-10-to-ORPHA mappings judged to be sufficiently specific and reliable were retained. Specifically, mappings were required to identify conditions in which the selected ORPHA code was as specific as possible, but no more specific than the corresponding ICD-10 code. Where multiple ICD-10 codes mapped to a single ORPHA code, all mapped ICD-10 codes used for case ascertainment are reported. Conditions were grouped according to ICD-10 chapter structure following Patrick et al. (2022), including separation of “Other Immune” conditions (D80–D89) from Chapter III; combination of Chapters V and VI as neurological diseases; and Chapters VII and VIII as Eye/Ear diseases. As noted by Patrick et al. (2022), many rare diseases are not precisely represented within ICD-10 coding systems and are therefore excluded by conservative mapping approaches of this type. Consequently, the total prevalence of rare diseases is likely to be underestimated. Furthermore, inaccuracies in clinical diagnosis and coding may result in some individuals annotated with a given ICD-10 code not having the corresponding condition.

Supplementary Information Table 1: Details of collection methods for selected characteristics collected in Health Survey England, UK Census and Our Future Health

Characteristic	Survey	Survey collection method	Our Future Health collection method
Ethnicity	UK Census for England, Wales and Scotland 2021/2022	Paper-based (2021/22) or online questionnaire (2022)<br><br>2021 England and Wales<br><br>'What is your ethnic group?'—multiple choice response; either 1) White OR 2) Mixed OR 3) Asian or Asian British* OR 4) Black or Black British OR 5) Chinese or other ethnic group<br><br>*Subgroups include Indian, Pakistani, Bangladeshi or any other Asian background<br><br>2022 Scotland<br><br>'What is your ethnic group?'—multiple choice response; either 1) White OR 2) Mixed OR 3) Asian, Asian Scottish or Asian British* OR 4) Black, Black Scottish or Black British OR 5) Other ethnic background<br><br>*Subgroups include Indian, Pakistani, Bangladeshi, Chinese or any other Asian background	Online questionnaire<br><br>"What is your ethnic group? Choose one option that best describes your ethnic group or background?" 1) White – English / Welsh / Scottish / Northern Irish / British OR 2) White – Irish OR 3) White – Gypsy or Irish Traveller OR 4) White – Polish OR 5) Any other White background OR 6) Mixed – White and Black Caribbean OR 7) Mixed – White and Black African OR 8) Mixed – White and Asian OR 9) Any other mixed/multiple ethnic background OR 10) Asian or Asian British – Indian OR 11) Asian or Asian British – Pakistani OR 12) Asian or Asian British – Bangladeshi OR 13) Chinese OR 14) Any other Asian / Asian British background OR 15) Black or Black British – African OR 16) Black or Black British – Caribbean OR 17) Any other Black / African / Caribbean background OR 18) Arab OR 19) Other OR 20) Prefer not to answer.
BMI	HSE 2021	BMI derived using weight (kg) and height (m) using the following formula; BMI = kg/m2	BMI was derived using weight (kg) and height (m) using the following formula; BMI = kg/m2
Height	HSE 2021	Verbal interview<br>Adjusted self-reported height derived from the following question: "Ask for an estimated height. How tall are you without shoes? You can tell me in metres or in feet and inches." Enter value either in 1) Metres OR 2) Feet and inches	In-person clinic measurement<br>Height in centimetres was measured using a Marsden HP-250P stadiometer. Participants confirmed they were happy to be measured and were asked to remove shoes, hats, and heavy clothing. They stood upright, looking straight ahead, while the head plate was lowered to the crown of the head. Height was recorded to the nearest centimetre. When footwear could not be removed, an estimate of shoe height was deducted from the measurement.
Weight	HSE 2021	Verbal interview<br>Adjusted self-reported weight derived from the following	In-person clinic measurement<br>Weight in kg was measured using SECA875 electronic scales, which

		question: “Ask for an estimated weight. How much do you weigh without shoes on? You can tell me in kilograms or in stones and pounds.” Enter value either in 1) Kilograms OR 2) Stones and pounds	record weight to one decimal place. Participants confirmed they were happy to be weighed and were asked to stand on the scale. Shoes and outer clothing were removed where appropriate; if this was not possible, weight was adjusted by deducting an estimated 1–2 kg for clothing.
WC	HSE 2021	Nurse appointment<br>Waist circumference in cm was measured using a tape with an insertion buckle at one end. Two measurements were taken. A third measurement was taken if the previous two measure differed by more than 3 cm. The mean of the two measurements (or two out of three that were closest together) was derived. Participants with a colostomy or ileostomy, or were pregnant or unable to stand were not measured	In-person clinic measurement<br>Waist circumference in centimetres was measured with a flexible tape measure. Participants confirmed they were happy to be measured and stood while the measurement was taken on bare skin or minimal clothing. The tape was placed around the midpoint between the lower ribs and the top of the hips, just above the navel, and the circumference was recorded to the nearest centimetre.
Smoking initiation	HSE 2021	Verbal interview<br>"How old were you when you started to smoke cigarettes regularly?" Enter Integer or enter 97 if 'Never smoked regularly'	Online questionnaire<br>Smoking initiation was derived from two questions:<br>“How old were you when you first started smoking on a regular basis?” Enter INTEGER OR 1) Do not know OR 2) Prefer not to answer<br>“How old were you when you first started smoking on most days?” Enter INTEGER OR 1) Do not know OR 2) Prefer not to answer
Alcohol consumption	HSE 2021	Verbal interview<br>Thinking now about all kinds of drinks, how often have you had an alcoholic drink of any kind during the last 12 months?’—multiple choice response; either 1) Almost every day OR 2) Five or six days a week OR 3) Three or four days a week OR 4) Once or twice a week OR 5) Once or twice a month OR 6) Once every couple of months OR 7) Once or twice a year OR 8) Not at all in the last 12 months	Online questionnaire<br>“About how often do you drink alcohol?” SELECT one of 7 from: 1) Daily or almost daily OR 2) Three or four times a week OR 3) Once or twice a week OR 4) One to three times a month OR 5) Special occasions only OR 6) Never OR 7) Prefer not to answer.
Smoking status	HSE 2021	Verbal interview<br>Smoking status derived from four questions. ‘May I just check, have you ever smoked a cigarette, a cigar or a pipe? We are referring here to tobacco cigarettes, not e-cigarettes or other vaping devices that use e-	Online questionnaire<br>Individuals who currently smoke cigarettes at any frequency (all/most days/occasionally) are considered current smokers. Individuals who do not currently smoke and have never smoked or smoked on fewer than 100 occasions in their lifetime are considered never smokers (including

		liquids.'— multiple choice response; either 1) Yes OR 2) No 'Do you smoke cigarettes at all nowadays?' multiple choice response; either 1) Yes OR 2) No 'Have you ever smoked cigarettes?' Multiple choice response; Either 1) Yes OR 2) No 'Did you smoke cigarettes regularly, that is at least one cigarette a day, or did you smoke them only occasionally?' Multiple choice response; Either 1) Smoked cigarettes regularly, at least 1 per day OR 2) Smoked them only occasionally OR 3) SPONTANEOUS: Never really smoked cigarettes, just tried them once or twice OR 4) SPONTANEOUS: Haven't stopped smoking	if they have smoked anything other than cigarettes). Individuals who currently do not smoke cigarettes but smoked on all or most days in the past or smoked on more than 100 occasions in their lifetime are considered former smokers. For version 1 questionnaires, based on variables: SMOKE_STATUS_1_1, SMOKE_TOBACCO_PREV_1_1, and SMOKE_100_TIMES_1_1. For version 2 questionnaires, based on variables: SMOKE_TOBACCO_TYPE_1_M, SMOKE_REG_1_M, SMOKE_STATUS_2_1, and SMOKE_100_TIMES_2_1.
E-cigarette use (vaping)	HSE 2021	Verbal Interview E-cigarette use was derived from the following question: "Have you ever used an electronic cigarette (e-cigarette), or any other vaping device? EXPLAIN IF NECESSARY: A vaping device is any product that you can use to inhale vapour rather like you would a cigarette. It includes ones that have a battery as well as ones that do not such as voke." Multiple choice question; either 1) Yes OR 2) No	Online questionnaire E-cigarette use was derived from two questions: (V1) "Have you ever used an electronic cigarette?"—choose one option: either 1) No OR 2) Yes OR 3) Prefer not to answer (V2) "Have you ever used any of these tobacco products, even once?" SELECT one of 8 from: 1) Cigarettes (manufactured or hand-rolled), 2) Electronic delivery devices that can be vaped, such as e-cigarettes (e.g., UWELL, Vype, Vuse, Vapouriz, WizMix), 3) Cigars, cigarillos or little filtered cigars (e.g., Montecristo, Romeo Y Julieta, Cohiba, Davidoff, Neos Red), 4) Chewing tobacco, snus, snuff, Gutkha or dip (e.g., Skruf, Tulsi, Sikandar, Conwood, Al Capone powder), 5) Shisha, hookah or water pipe, 6) Tobacco pipe, 7) I have not used any of these tobacco products, and 8) Prefer not to answer.
Current self-reported health status	HSE 2021	Verbal questionnaire The variable was derived from following question: "How is your health in general? Just tell me the number beside the row that best applies". Multiple choice response; either 1) very good OR 2) good OR 3) fair OR 4) bad OR 5) very bad	Online questionnaire "In general, how would you rate your overall health SELECT one of 4 from: 1) Excellent OR 2) Good OR 3) Fair OR 4) Poor OR 5) Do not know OR 6) Prefer not to answer.

Any long-standing physical or mental health conditions or illnesses	HSE 2021	Verbal questionnaire "Do you have any physical or mental health conditions or illnesses lasting or expected to last 12 months or more?"—multiple choice response; either 1) Yes OR 2) No	Online questionnaire "Do you have any physical or mental health conditions or illnesses lasting, or expected to last, 12 months or more?"—SELECT one of 4 from: 1) Yes OR 2) No OR 3) Do not know OR 4) Prefer not to answer.
Physical activity	HSE 2021	Verbal questionnaire "During the past four weeks, on how many days did you do a walk of at least 10 minutes? (That is since <*date of interview – 4 weeks*>)" Enter Integer in range 1-28	Online questionnaire "Thinking about the last 4 weeks, in a typical WEEK, on how many days did you walk for at least 10 minutes at a time? (Include walking that you do at work, travelling to and from work, and for sport or leisure)" Enter INTEGER [Require ≥ 0, ≤ 7Units: days] OR 2) Do not know 3) Unable to walk OR 4) Prefer not to answer

HSE, Health Survey England; BMI, body mass index; WC, waist circumference. For further information about HSE see[40]; V1, questionnaire version 1; V2, questionnaire 2. Questions use to create smoking status-related variables in OFH are omitted for presentation purposes; for details of each question see OFH Questionnaire data: https://ourfuturehealth.gitbook.io/our-future-health/data/questionnaire-data

Supplementary Information Table 2: Medication usage variable reference

Variable	Description	Abbreviation
derived.medicates_auto	Autoimmune / inflammatory medications (e.g., DMARDs, TNF inhibitors)	AUTO meds
derived.medicates_bone	Bone health / osteoporosis medications (e.g., bisphosphonates, HRT, vitamin D)	MSK meds
derived.medicates_cancer	Cancer / oncologic treatments (e.g., chemotherapy, immunotherapy)	CANC meds
derived.medicates_diab	Diabetes / metabolic medications (e.g., insulin, metformin, GLP-1s)	DIAB meds
derived.medicates_digest	Gastrointestinal medications (e.g., PPIs, laxatives, IBD medications)	GI/LIVER meds
derived.medicates_endo	Endocrine disorder medications (e.g., thyroid, pituitary, adrenal)	ENDO meds
derived.medicates_cvd	Heart or circulatory medications (e.g., statins, antihypertensives)	CVD meds
derived.medicates_resp	Lung or respiratory medications (e.g., inhalers, corticosteroids)	RESP meds
derived.medicates_mental	Mental health medications (e.g., antidepressants, antipsychotics)	PSYCH meds
derived.medicates_neuro	Neurological medications (e.g., anti-epileptics, Parkinson's, migraine)	NEURO meds
derived.medicates_pain	Pain relief medications (e.g., NSAIDs, opioids, paracetamol)	PAIN meds
derived.medicates_repro	Reproductive / sexual health medications (e.g., HRT, contraception, erectile dysfunction medications)	REPRO meds
derived.medicates_supp	Supplements / vitamins / minerals (e.g., vitamin D, calcium, fish oil, iron)	SUPP meds
derived.medicates_cvd_diab	Cardiometabolic medication use (CVD + diabetes combined)	CVD/DIAB meds
derived.medicates_mental_pain	Mental health or pain medication use (PSYCH + PAIN combined)	PSYCH/PAIN meds
derived.medicates_auto_end	Immune / inflammatory system medication use (AUTO + ENDO)	AUTO/END meds
derived.medicates_supp_msk	Supplements / bone / nutritional medication use (SUPP + MSK)	SUPP/MSK meds
derived.num_meds_domains	Number of medication domains used	#DOMAINS
derived.any_meds_flag	Any medication use	ANYMeds
derived.polypharmacy_flag	Polypharmacy (≥3 medication domains)	POLYPHARM
derived.prop_systems_used	Proportion of medication system groups used	PROPSYS
derived.multi_med_system_use	Use across ≥2 medication system groups	MULTISYS

Supplementary Information Table 3: ICD-10 Codes used to determine EHR diagnoses

Condition	ICD-10 Codes used	Description
Cancer	C	Malignant neoplasms (C00–C97; prefix match)
Asthma	J45, J46	Asthma and status asthmaticus
Type 2 Diabetes	E11	Type 2 diabetes mellitus
Depression	F32, F33	Depressive episodes and recurrent depressive disorder
Hypertension	I10	Essential (primary) hypertension
High Cholesterol	E78	Disorders of lipoprotein metabolism and other lipidaemias
Osteoarthritis	M15, M16, M17, M18, M19	Polyarthrosis and osteoarthritis of specific joints
